# Computer Vision Applications in Intelligent Transportation Systems: A Survey

**DOI:** 10.3390/s23062938

**Published:** 2023-03-08

**Authors:** Esma Dilek, Murat Dener

**Affiliations:** Department of Information Security Engineering, Graduate School of Natural and Applied Sciences, Gazi University, 06560 Ankara, Turkey

**Keywords:** intelligent transportation systems, computer vision, automatic number plate recognition (ANPR), traffic sign detection, vehicle detection, pedestrian detection, lane line detection, obstacle detection, anomaly detection, structural damage detection, autonomous vehicles

## Abstract

As technology continues to develop, computer vision (CV) applications are becoming increasingly widespread in the intelligent transportation systems (ITS) context. These applications are developed to improve the efficiency of transportation systems, increase their level of intelligence, and enhance traffic safety. Advances in CV play an important role in solving problems in the fields of traffic monitoring and control, incident detection and management, road usage pricing, and road condition monitoring, among many others, by providing more effective methods. This survey examines CV applications in the literature, the machine learning and deep learning methods used in ITS applications, the applicability of computer vision applications in ITS contexts, the advantages these technologies offer and the difficulties they present, and future research areas and trends, with the goal of increasing the effectiveness, efficiency, and safety level of ITS. The present review, which brings together research from various sources, aims to show how computer vision techniques can help transportation systems to become smarter by presenting a holistic picture of the literature on different CV applications in the ITS context.

## 1. Introduction

Smart city technologies are an important element of effectively managing the rapid industrialization of the world today, as they can help to address the economic and environmental problems resulting from the increase in urban populations. Smart cities, which integrate traditional infrastructure and public services with technology to create a more efficient, sustainable, and accessible system while meeting the needs of city residents, also transform the traditional understanding of city management. Intelligent transportation systems (ITS), which are among the key components of smart cities, are developed to improve transportation safety and mobility, reduce environmental impact, promote sustainable transportation development, and enhance productivity [1].

ITSs offer modern solutions to transportation-related problems, such as traffic jams and accidents, and help to ensure the safety of road users by utilizing data collected from surrounding vehicles, infrastructure, and other networks. ITS applications exist in a variety of forms, including collaborative highway maneuvers, sharing road safety information, optimization of traffic signals, and autonomous driving [2]. ITS, which can be defined as integrated transportation management systems consisting of advanced data communication, information processing, and traffic management technologies, can instantly process real-time data collected from heterogeneous sources and analyze it to support better decision making [3].

Decisions that were formerly made based on human experience can now be made using computers by digitizing information. Moreover, predictions and forecasting can also be improved through the use of new-generation artificial intelligence (AI) algorithms. Thanks to AI technologies, it is possible to develop systems that can make decisions based on data. These technologies have also led to radical changes in many areas, including public transportation and transportation systems, and have helped to make different modes of transportation safer, greener, smarter, and more efficient [4]. Yuan et al. [5] divide AI applications in the field of ITS into three main categories, namely (i) detection/recognition, (ii) prediction, and (iii) management. Machine learning (ML) methods, a sub-branch of AI, act as the brain function of ITS and determine the accuracy, reliability, and smartness of the systems. In particular, in recent years, it has been observed that deep learning (DL) methods, which are a subset of ML methods, are being effectively utilized in classification and prediction works in different areas of ITS [3].

Computer vision (CV) is an AI field that enables machines to derive meaningful information from digital images, videos, and other visual inputs, as well as to act based on this information [6]. CV, in which both ML and DL methods are used, addresses image and video processing problems and offers solutions that can be used in the process of automating transportation systems and making them safer. CV techniques are actively used in various ITS applications, such as automatic license plate detection and recognition, traffic sign detection and recognition, vehicle detection and classification, pedestrian detection, obstacle and lane line detection, anomaly detection in video surveillance cameras, vehicle and passenger tracking, structural damage detection, and autonomous vehicle applications. CV methods are appealing in these applications largely due to their cost-effectiveness, as well as the wide range of applications that CV can support [7].

**Figure 1:**
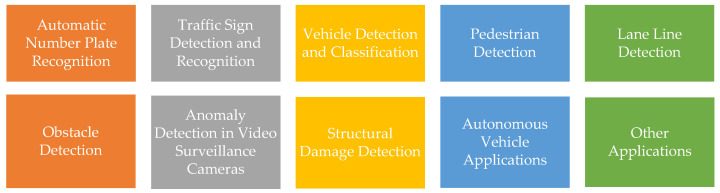
Computer Vision Applications in Intelligent Transportation Systems.

Within the scope of this survey, CV methods used in ITS are categorized and examined under 10 headings, as shown in Figure 1. Specifically, this survey examines CV applications used in ITS and proposes CV studies that could be conducted in the future to increase the efficiency of ITS. Since it will not be possible to cover all existing literature on CV studies in the field of ITS, a detailed analysis and an examination are performed as part of this survey, and a representative subset of current approaches is selected. To the best of our knowledge, this survey is the most recent work to investigate CV in ITS from a holistic and inclusive perspective within the last decade. The main contributions of this survey to the literature can be summarized as follows:

- CV applications in the field of ITS, along with the methods used, datasets, performance evaluation criteria, and success rates, are examined in a holistic and comprehensive way.
- The problems and application areas addressed by CV applications in ITS are investigated.
- The potential effects of CV studies on the transportation sector are evaluated.
- The applicability, contributions, shortcomings, challenges, future research areas, and trends of CV applications in ITS are summarized.
- Suggestions are made that will aid in improving the efficiency and effectiveness of transportation systems, increasing their safety levels, and making them smarter through CV studies in the future.
- This research surveys over 300 studies that shed light on the development of CV techniques in the field of ITS. These studies have been published in journals listed in top electronic libraries and presented at leading conferences. The survey further presents recent academic papers and review articles that can be consulted by researchers aiming to conduct detailed analysis of the categories of CV applications.
- It is believed that this survey can provide useful insights for researchers working on the potential effects of CV techniques, the automation of transportation systems, and the improvement of the efficiency and safety of ITS.

The remainder of this survey is organized as follows. In Section 2, the evolution of CV in the field of ITS is discussed, along with preliminary information. In Section 3, CV applications in ITS and the methods used in the literature are investigated. In Section 4, the applicability, contributions, deficiencies, and difficulties of CV applications in ITS are discussed, and suggested solutions to existing problems are presented. In the fifth and final section, research results and directions for future research are provided.

## 2. Computer Vision Studies in the Field of ITS

### 2.1. Evolution of Computer Vision Studies

While there are many methods used in CV studies in the literature, the methods most commonly used in the field of ITS are summarized in the following sections.

#### 2.1.1. Handcrafted Techniques

Early CV researchers focused primarily on the use of different handcrafted spatiotemporal features and traditional image-processing methods [8]. Handcrafted features are those obtained with the help of several algorithms using the information that exists in the image itself. These features have been widely used in previous works using traditional ML approaches for object recognition.

Deformable part-based models, integral channel features (ICF), aggregated channel features (ACF), histograms of oriented gradients (HOG), local binary patterns (LBPs), scale-invariant feature transform (SIFT), Gabor filters, local ternary patterns (LTPs), local phase quantization (LPQ), rotation-invariant co-occurrence local binary patterns, completed local binary patterns, rotated local binary pattern images, and globally rotation-invariant multi-scale co-occurrence local binary patterns are among the handcrafted techniques that were used to extract features from images in previous studies [9]. Newer approaches, such as convolutional neural networks (CNNs), do not require such handcrafted features, as they can learn features from the image data.

#### 2.1.2. Machine Learning and Deep Learning Methods

Machine learning, one of the most prominent subfields of AI, deals with the design and creation of algorithms for the recognition of complex patterns and decision making based on experimental data [10]. Problems handled with ML methods can be broadly categorized into (i) supervised, (ii) unsupervised, and (iii) reinforcement learning methods. In supervised learning, the goal is to estimate an output by taking feature vectors as inputs. Here, the ML algorithms establish a temporary model between the input and output values. The model attempts to estimate the output of the test data, which it has never seen before. If the ML model divides the input data into certain categories, then it is considered to be a classification; if the model tries to find continuous values using input values, then it is considered to be a regression. For both problems, the data must be labeled beforehand. The most frequently used algorithms for classification are support vector machine (SVM), collective/ensemble learning, k-nearest neighbors, and random forest (RF). Support vector regression and Gaussian process regression models are used in the literature for regression. Supervised learning models have been used for the classification of vehicles [11,12], classification of traffic lights [13], recognition and classification of license plate characters [14,15,16,17], detection of traffic signs [18], detection of pedestrians [19,20], etc.

Since assigning labels to millions of data points is a laborious and inefficient process, unlabeled data can be grouped through the use of unsupervised learning algorithms. Using different mathematical infrastructures, these algorithms classify data according to their own criteria. Among the unsupervised learning algorithms, methods such as k-means, density-based spatial clustering of applications with noise (DBSCAN), and the Gaussian Mixture Model (GMM) are used to identify groups and clusters. Unsupervised learning models have been used for the recognition of license plates [21], detection of obstacles [22], detection of road cracks [23], etc.

Based on the idea that there may be no available training data in some cases, reinforcement learning models have been developed, inspired by the knowledge acquisition processes of infants. These algorithms utilize a type of learning that tries to find the steps that a subject (a robot, an autonomous vehicle, etc.) must perform in order to receive the highest reward in the environment. Subjects working according to the reward–punishment mechanism perform actions in an attempt to understand the environment. After a range of these actions have been performed, the steps that lead to the highest reward score are saved, and these turn into behaviors. There are studies in the literature in which reinforcement learning methods were used in traffic signal control systems [24], traffic timing applications [25], and for the detection of lane lines [26].

**Figure 2:**
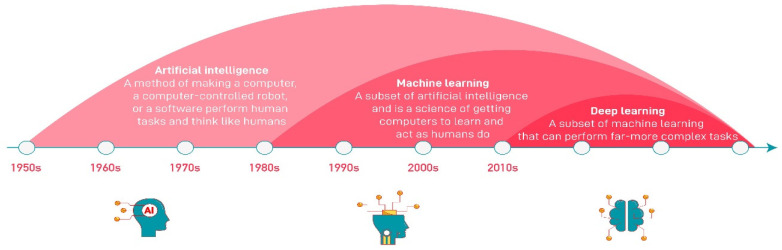
Evolution of Artificial Intelligence, Machine Learning, and Deep Learning.

Although traditional ML methods such as SVM [16,19,27], Bayesian networks [28], and the Kalman filter (KF) [29,30] were used in early ITS research [3], the problem-solving capabilities of algorithms have improved over time due to the development of hardware resources and the increasing amount of training data. As can be seen in Figure 2, while the concept of ML was dominant in the years between 1980 and 2010 [31], it was observed that these classical ML algorithms had difficulties processing large amounts of data; in response, artificial neural network (ANN)-based models began to emerge. However, since classical ANN models are insufficient for processing big data, modern ANN structures have been developed, which led to the development of DL models. While models that extract features from images and those that perform classification using these features are separate in ML algorithms, DL models can perform both processes in a single artificial neural stack. 

#### 2.1.3. Deep Neural Networks (DNNs)

A DNN consists of multiple layers of ANN architectures and DNN models. It contains an input layer, one or more hidden layers, and an output layer. As a groundbreaking innovation, DNNs have produced satisfactory results on basic tasks such as the classification, detection, and segmentation of objects. Thus, AI technologies have become important in the field of ITS thanks to DNNs.

There are many types of DNN models which are used for different purposes. For example, deep belief networks (DBN) have been used for facial recognition [32] and crack detection [33]; stacked auto-encoder (SAE) networks have been used for object detection [34], image compression [35], and video retrieval [36]; restricted Boltzmann machines (RBM) have been used for face recognition [37], and YOLO (You Only Look Once)-based DL methods have been utilized in object-detection [38] tasks.

#### 2.1.4. Convolutional Neural Networks (CNNs)

In the field of CV, the DNN most widely used to extract features from images is the CNN. In essence, CNNs try to imitate the working principles of the human brain and visual cortex, making use of multiple layers to recognize objects. One of the outstanding strengths of CNNs is their ability to classify objects into thousands of classes. Other advantages of CNNs include their relative robustness to image noise, along with their robustness to rotation and changes in the position of objects in an image. Their biggest disadvantages are their long training time and the need for a large training dataset [39]. The use of graphics cards and parallel processors during training contributes positively to the training and classification time of CNN models.

**Figure 3:**
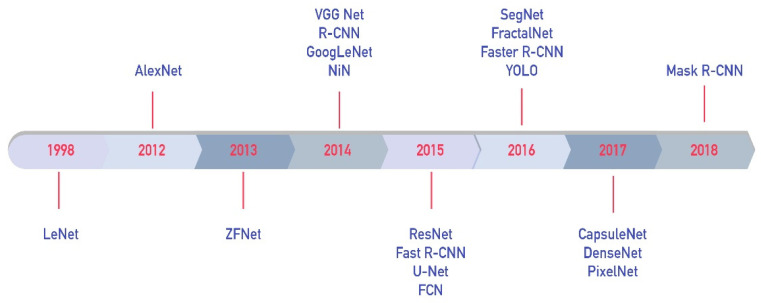
CNN Architectures.

Variants of CNN networks are widely used in CV studies in the field of ITS. There are a number of CNN-based studies in the literature, such as those focused on automatic license plate recognition [40,41], traffic sign detection and recognition [25,42,43,44,45,46,47,48,49,50,51], vehicle detection [52,53,54,55], pedestrian detection [56,57,58,59,60], lane line detection [61,62,63], obstacle detection [64], video anomaly detection [65,66,67,68], structural damage detection [69,70,71,72,73,74,75,76,77,78], and steering angle detection [79,80,81,82] in autonomous vehicles. The most popular and advanced CNN-based architectures in the literature [83,84] are presented in Figure 3.

#### 2.1.5. Recurrent Neural Networks (RNNs)

RNNs are specially designed for modeling sequence data. The RNN is a powerful DL method, as it can directly learn the mapping between input and output sequences. However, traditional RNNs are impacted by the gradient vanishing problem. Long short-term memory (LSTM) networks were developed to solve this problem. An LSTM network is a type of RNN that can learn order dependence in sequence prediction tasks. In LSTM networks, memory cells are designed to maintain their state over time and learn long-term dependencies. RNNs have been used for license plate recognition [85], lane line detection [63], and crack classification [76] tasks, as well as in autonomous vehicle applications [86].

The gated recurrent unit (GRU) is a simplified variant of LSTM that does not contain discrete memory cells. The GRU is faster to train, while retaining its resilience to the vanishing gradient problem.

Convolutional LSTM networks have been used for the detection of anomalies in videos [87,88,89,90,91,92], as well as in autonomous vehicle applications [86,93], while a convolutional GRU network was used for video anomaly detection [94].

#### 2.1.6. Generative Adversarial Networks (GANs)

The GAN is an approach based on generative modeling that uses DL methods to produce high-quality images. In recent years, GANs have been widely studied by DL communities in the context of video anomaly detection studies.

Generative modeling is an unsupervised learning method that involves automatically discovering and learning regularities or patterns in the input data, which the model can use to generate or create new examples that may be reasonably drawn from the original dataset. GANs are based on a learning approach that utilizes two sub-models, called the discriminator and generator, to train generative models. GAN is based on the idea of training implicitly through the discriminator, which is an ANN that dynamically updates itself and can gauge how realistic the input appears. Rather than minimizing the difference from a particular image, the generator learns in an unsupervised manner to fool the discriminator. GANs have been widely used in recent video anomaly detection studies [95,96,97,98,99].

#### 2.1.7. Other Methods

Hybrid methods include a combination of multiple ML or DL methods used in CV techniques. There are many intelligent transportation applications for this approach, such as license plate recognition [85,100,101], video anomaly detection [68,89,92,102], automatic license plate recognition [25,103], vehicle detection [11,12,53,55], pedestrian detection [58,104], lane line detection [63,105], obstacle detection [106,107,108,109,110], structural damage detection [111,112,113], and autonomous vehicle applications [13,114,115].

Vaswani et al. [116] introduced an encoder–decoder architecture based on attention layers, named the transformer. A transformer neural network takes an input sentence in the form of a sequence of vectors, converts it into a vector called an encoding, and then decodes it back into another sequence. An essential part of the transformer is the attention mechanism, which represents how important other tokens in an input are for the encoding of a given token. Transformers are used for image classification, object detection, and image compression in CV applications. In the field of ITS, they have been used in license plate recognition [85], pedestrian detection [117], and driver distraction detection [118] studies.

### 2.2. Computer Vision Functions

**Figure 4:**
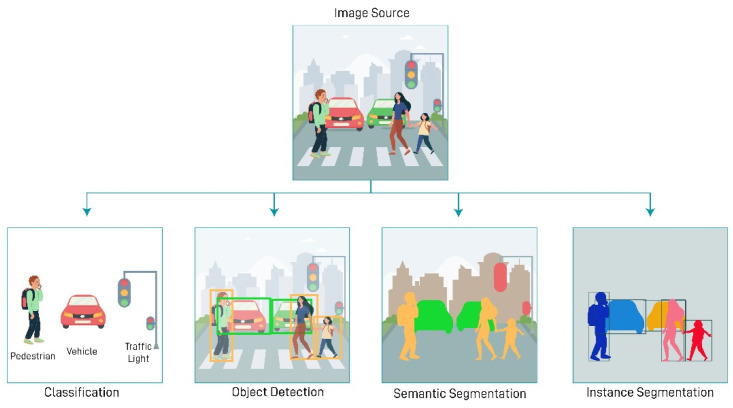
Basic Functions Performed by Computer Vision Techniques in the Field of ITS.

Among the data emerging in the field of ITS, visual data are among the most voluminous kind. CV studies enable the analysis of both images and videos and provide detailed information about the traffic situation. Figure 4 presents some of the basic functions performed by CV techniques in the field of ITS. As can be seen from the figure, CV methods play a significant role in performing basic functions such as (i) classification, (ii) object detection, (iii) semantic segmentation, and (iv) instance segmentation [119].

Object classification can be performed by using CV techniques to process the image or video data obtained by the cameras. A label can be assigned automatically to each sub-object in the image. To achieve this, the objects are divided into parts and given to the model.

Another function performed using CV techniques is object detection. The detection of traffic objects such as vehicles and pedestrians in an image plays a vital role in the development of many applications. Important functions, such as detecting traffic density, detecting pedestrians that suddenly appear on the road, or detecting the locations of other vehicles for autonomous driving vehicles, can be performed with DL-based object detection models. The main feature that distinguishes object detection from classification is that the former can determine the coordinates of the area in which it is located, in addition to classifying each relevant object in the image. AI models of this kind can perform both classification and regression. The object with corner coordinate points becomes positionable by the machine in the image.

In the semantic segmentation context, all pixels belonging to objects are classified. As can be seen in Figure 4, cars are automatically marked in blue and pedestrians in red by CV techniques. Grouping all pixels of the object and assigning the appropriate class to each is a challenging problem. Semantic segmentation models assign the same groups of objects to a single class. However, vehicles and pedestrians in traffic sometimes need to be grouped individually. Under these circumstances, instance segmentation methods are used. The purpose of instance segmentation, like semantic segmentation, is to assign classes to pixels. With instance segmentation, objects belonging to the same class can be grouped separately, even if they overlap.

A framework outlining which problems in the field of ITS can be solved with CV techniques adapted from [120] is presented in Table 1.

## 3. Computer Vision Applications in Intelligent Transportation Systems

ITSs have made many contributions to transport systems, including improving transport safety, increasing transport system efficiency, aiding law enforcement, and boosting energy conservation and emissions reduction. CV applications play an important role in this context and are thus of interest to researchers. In the Web of Science (WoS) database, there are more than a thousand studies that have been published in the field of CV in ITS since 2000. Since the field of ITS is multi-disciplinary, it has been observed that these publications extend across multiple scientific publication categories, such as electrical/electronic engineering, computer science, transportation science technology, civil engineering, telecommunications, and automation control systems.

Research into the use of CV methods in road transport systems was presented in [7] and [121], while a comprehensive review of traditional CV techniques for traffic analysis systems with a particular focus on urban environments was presented in [122]. However, those studies lack the state-of-the-art CV methods developed within the last decade. ML techniques have been used effectively to make transportation systems more efficient, especially in recent years, in. In current research, it has been noted that traditional ML models are now being replaced by new learning techniques and that DL techniques are widely used in ITS. A comprehensive study focusing on the use of DL models to increase the intelligence of transportation systems was presented by Wang et al. in [3]. Authors explored the use of DL models in various transportation applications including (i) traffic sign recognition, (ii) traffic flow prediction, (iii) traffic speed prediction, and (iv) travel time prediction. Applicability and shortcomings of DL models in the context of ITS and evolving future trends were also argued.

It is predicted that transportation systems will become smarter through the use of ML, big data analysis, and more powerful computing resources [3]. In the following sections, the studies in the literature on various CV applications in ITS that are listed in Figure 1 are categorized and summarized. The studies in each category are also presented in a table at the end of each section with major highlights. When analyzing CV applications in ITS, it becomes clear that there are a number of techniques employed in the literature for different purposes, as well as various datasets and performance metrics used to measure the successes of the proposed methods. This use of different datasets and performance metrics makes it difficult to analyze the performance of a given method and compare it to that of other methods. Moreover, this may also cause it to appear that there was a decrease in the performance of methods year by year in some categories. For this reason, the performance of methods in each category is accompanied with the datasets and metrics used in the literature.

### 3.1. Automatic Number Plate Recognition (ANPR)

ANPR systems, which enable traffic management and instant traffic monitoring and contribute to the collection of important statistics on road conditions, were among the first CV applications in the field of ITS. ANPR technology provides the ability to detect and recognize vehicles based on their license plates (also known as number plates) without human intervention using recognition techniques.

The ability to track vehicles with known license plates makes it possible to track vehicles in urban areas, count vehicles, detect vehicles, determine average traffic flow rates, detect the movement directions of vehicles, detect traffic violations, find wanted vehicles, and enforce the law. ANPR technologies offer diversified smart transportation solutions, such as access control, automatic calculation of highway or parking usage fees, estimation of queue lengths, and congestion pricing.

In ANPR systems, license plate images are obtained from the intended scene by means of a camera. Still images or video frames are first captured, after which license plates are obtained from the captured images by applying alphanumeric transformations using a series of image-processing-based recognition algorithms. A typical ANPR system comprises the following processes: (i) general image acquisition, (ii) number plate extraction (NPE), (iii) character segmentation (CS), and (iv) character recognition (CR).

ANPR systems include complex optical, computing, and digitizing processes. OCR (optical character recognition) engines are often optimized for specific countries, as current ANPR products do not offer a standardized solution for all countries. An ANPR system developed for one country will not function effectively in another country, meaning that each system must be designed according to the region in which it is deployed. Since each ANPR solution has its own strengths and weaknesses, these solutions must be optimized according to the needs of the regions in which they will be used [123].

Various factors adversely affect the performance of ANPR systems, such as the license plate’s physical situation, non-standardized formats, complex scenes, camera quality, camera mounting position, tolerance to distortion, motion blur, contrast issues, reflections, rendering and memory limitations, environmental conditions, indoor/outdoor or day/night conditions, software tools, and/or other hardware-based restrictions. These difficulties encountered in ANPR technologies make this field interesting for researchers [123].

An improved SVM-based algorithm was proposed in [14] for challenging and complex plates, and a self-learning algorithm based on Bayesian probability and Levenshtein text-mining, which can improve the matching accuracy of the ANPR system, was proposed in [124].

The accuracy rates of ANPR systems can be significantly improved if the camera is set up correctly, considering distance, tilt angles, region of interest (ROI), zoom level, and lighting factors. Processing capabilities vary depending on the environment and camera shutter speed. A 98% recognition rate was obtained in [125], where HD (high-definition) cameras were used. In [126], which tested the model in real time using HD cameras and a dataset containing more than 2790 characters, a recognition rate of 99.5% was achieved with a similar system. This study employed the connected component analysis (CCA) technique, which uses an OCR algorithm for Qatar’s license plate format. However, the technique proposed in this study was computationally expensive and impacted by memory and processing time constraints, as well as high system costs. Although high recognition rates can be achieved with HD camera systems, these systems are computationally costly.

In [40], CNN-based algorithms and a YOLO object detector were applied in real-time scenarios for Brazilian license plate extraction, with success rates varying between 98.33% and 100% on different tested datasets. In [127], a scale-adaptive model was applied to more than 2600 mixed-format license plates and tested in real-time scenarios, achieving an overall success rate of 97%. However, the proposed method requires extensive model training to handle changing situations.

A real-time method of detecting license plates from video streams using a CNN-architecture-based DL approach was proposed in [41]. In this method, license plates could be extracted from images with an accuracy of 91%, the character recognition success rate was 93%, and license plate recognition from real-time video streams with an average accuracy of 85% was achieved.

Classifiers have been used in some ANPR studies; in many cases, a combination of multiple classifiers or multi-stage classification schemes were used in parallel. An ANN for ANPR was used in [128]. In [40], a CNN was used in a real-time scenario, achieving good results for each phase of the ANPR system. Neural-network-based methods seem to be promising solutions for ANPR systems, and have been utilized in a number of studies, including [21,85,129,130,131,132,133].

An online license plate detection and recognition method for vehicles in motion in parking areas was proposed in [100]. In this study, which evaluated three different models (namely HAAR Cascade/CNN, OpenCV2, and YOLOV3/OpenCV3), it was observed that the model in which YOLOV3 and OpenCV3 were used together drew a bounding box around the license plates with 100% accuracy and could recognize the characters on license plates with 95% accuracy.

In the study reviewed in [134], an automated vehicle tracking system incorporating experimental CV techniques for real-time license plate recognition was proposed to provide access control for vehicles and increased security for an academic institution. A vehicle monitoring framework was designed that employed different technologies and tested different camera angles. The effect of environmental changes on the accuracy of the OCR application was evaluated. The design science research methodology was followed to develop the vehicle tracking framework. Image enhancement algorithms were tested with the goal of discovering the most suitable options. It was demonstrated that a cost-effective solution could be provided by utilizing the existing camera infrastructure and appropriate license plate recognition software technologies in the academic institution, achieving 96% success under the optimum working criteria established for the vehicle tracking framework.

In [101], an efficient DL-based vehicle license plate number recognition (DL-VLPNR) model was proposed to identify and analyze a vehicle’s license plate characteristics. In the proposed method, faster region-based CNN (Faster R-CNN) with an Inception V2 model was used to detect alphanumeric characters on the license plate of a vehicle in an image. Subsequently, the characters on the detected plate were extracted with the Tesseract OCR model, and the performance of the DL-VLPNR model was verified using the FZU Cars and HumAIn2019 datasets. The results were analyzed to assess different criteria, such as precision, recall, F1 score, accuracy, and mAP (mean average precision). Experimental results showed good detection and recognition performance for the DL-VLPNR model, with an accuracy of 98.6%.

Tesseract is the most widely adopted OCR engine, thanks to its ability to recognize over 100 languages; it can also be trained on new or unlisted languages. Most ML-based ANPR software developers use this engine for their vehicle recognition applications. Using a tested dataset of approximately 1300 images, Tesseract’s OCR and local binary pattern extraction methods were applied in [135] for real-time scenarios, and an overall accuracy of 96.73% was achieved. Notably, since only fixed angles were considered for image acquisition in this study, there is a need to investigate the same algorithms from different angles. In [101], the authors also used the Tesseract OCR model for plate extraction and achieved high ANPR accuracy.

A detailed review of ANPR algorithms was conducted by Mufti and Shah [123], who presented a performance comparison of the techniques and datasets used in ANPR systems as well as advancements and challenges faced. In [136], Joshi et al. investigated automatic license plate detection and recognition methodologies in studies published between 2016 and 2020.

In the research report compiled by Shashirangana et al. in [137], approaches and techniques used in automatic license plate recognition solutions in the current literature were investigated and analyzed. The report observed that while single-stage DL-based solutions achieved a high performance on various datasets, multi-stage object-detection-based DL solutions can be pre-trained on large datasets but will have lower computational efficiency and accuracy than single-stage approaches. The article carried out a comprehensive comparison of related studies and listed the requirements for benchmark datasets in practice. Additional information was also presented regarding the open challenges faced by researchers and future research topics for ANPR solutions. The authors pointed out that while single-stage DL-based methods perform well with various datasets, multi-stage object-detection-based DL methods yield lower accuracy rates and computational efficiency, but they can be pre-trained on large datasets.

Table 2 presents the list of ANPR studies using CV methods in the literature. Further information can be found in [123].

As can be seen from Table 2, AI-based ANNs have been used in recent ANPR studies, and developments in these technologies have also improved the performance of ANPR systems. In recent studies, it is recognized that CNN-based AI architectures are preferred in ANPR solutions, and that recognition performance is improved through the use of CNN variant methods such as YOLOv3, Faster R-CNN, and Inception V2. The literature shows that automatic license plate recognition rates of up to 100% can be obtained by using the YOLOv3 method. However, it is also observed that a wide variety of datasets, which differ depending on the countries in which they are employed, are used to measure the performance of the developed ANPR methods. Moreover, in some studies, the authors prefer to measure the performance of their methods by producing their own datasets.

### 3.2. TrafficSign Detection and Recognition

Traffic sign recognition, which is used in autonomous vehicles and advanced driver assistance systems (ADAS), is a type of CV application that aims to identify the traffic signs in an image from a limited number of options. Essentially, this is a classification task. More specifically, traffic sign recognition is an image classification problem, and its accuracy is evaluated with reference to the correctly classified part of the images. Traffic sign detection, which is a similar task, involves identifying the region of the image that contains a traffic sign. The accuracy of traffic sign detection is measured in terms of mAP; moreover, to determine whether a detected region is correct, the intersection over union value (IoU) is calculated and compared with a threshold value, usually set to 0.5 [47].

Traffic sign recognition is a difficult task due to the impact of numerous factors, such as angle differences, lighting conditions, blurring, partial shading, color distortion, and contrast deterioration of the images used in the recognition of traffic signs.

A typical image detection/classification process consists of (i) the feature extraction stage, in which summary information is extracted from the image, followed by (ii) the classification stage, in which recognition is performed. In the traffic sign recognition process, feature extraction and classifier selection in pattern recognition are among the factors that affect the accuracy rate. For these operations, different algorithms have been tested to find the one most suitable for solving the problem. The classical ML approach involves the classification of features using algorithms such as SVM and RF; however, these algorithms were found to be insufficient to handle real-life events [141,142]. It has been observed that various types of discriminative and representative features have been adopted in previous studies for the traffic sign recognition task. For example, Ruta et al. [141], Dalal and Triggs [143], and Liu et al. [144] used HOG and Haar wavelets for feature extraction in traffic sign recognition. The SVM ML method for classical traffic sign classification has been widely used by authors including Greenhalgh and Mirmehdi [15], Maldonado-Bascón et al. [16], Lafuente-Arroyo et al. [17], and Le et al. [27]. For feature classification, k-dimensional (k-d) trees and RF classifiers seem to be preferred by authors including Zaklouta et al. [142] and Zaklouta and Stanciulescu [145].

Table 3 lists some of the traffic sign recognition studies in the literature that employ traditional ML methods. As the table shows, the highest accuracy rate that can be obtained using the German Traffic Sign Recognition Benchmark (GTSRB) dataset with traditional ML methods such as RF is 97.2%.

Following the emergence of DNN models after 2012, handcrafted techniques and traditional ML methods were replaced by DL methods in the literature, with the latter providing higher accuracy rates in recent traffic sign detection/recognition studies. In traffic sign recognition and classification applications, it is considered that DL models can be applied provided that they can be formulated as a regression or Markov decision process (MDP) problem and that a large amount of training data are available or can be collected at low cost [3].

The studies carried out by Ciresan et al. [42] and Sermanet and LeCun [43] are among the first in the literature to employ a CNN DL method for traffic sign recognition. In [44], preprocessing steps such as image translation, rotation, and scaling were applied to prevent overfitting and improve the generalization performance of the system. The hinge loss stochastic gradient descent (HLSGD) technique was used by Jin et al. [45] to improve the training time of the CNN network. This model also achieved higher accuracy compared to previous studies. In [46], Haloi proposed a spatial transformer layer in the input feature map that included (i) a localization network, (ii) a grid generator, and (iii) a sampling unit to make the traffic sign recognition task robust against image skew, rotation, clipping, and scaling operations. In addition, a modified GoogLeNet was used as an inception module, with various sizes of convolutional filters that were used to better capture the features of different abstractions [46].

Traffic sign detection and traffic sign recognition were handled together by Qian et al. [47]. First, using the R-CNN variant, potential traffic sign regions in the images were determined by means of RGB space thresholding; subsequently, the traffic sign recognition process was carried out using the CNN model. System performance was evaluated by mixing the GTSRB traffic dataset with the MNIST [148] and CASIA datasets [149]. Traffic sign detection and recognition tasks were also explored by Changzhen et al. [48] using Chinese traffic signs, following the approach suggested by the authors in [47].

RBM and canonical correlation analysis (CAA) [150] were used by Li and Yang [18] and Li et al. [25] for feature extraction. After applying preprocessing steps such as drizzling, gray-scale normalization, and size normalization, low-level features such as LBPs were extracted. Two-layer RBM was used to convert low-level features to high-level features, after which the relationship between canonical variables was determined by applying CAA. In the last step, feature vectors were classified using the SVM ML method. Using a modified R-CNN framework, Li et al. [25] identified and classified US traffic signs with a DL model that incorporated cuda-convnet.

Real-time traffic sign recognition, an important requirement for autonomous vehicles, was studied by Jung et al. [49]. In this study, using a simple color segmentation method, the model processed an average of 16.9 frames per second (fps) to quickly detect regions containing possible traffic signs. In a departure from previous studies, Zeng et al. [50] opted to use the lab-based perceptual color space rather than the RGB color space and obtained higher accuracy in traffic sign recognition. Using a network with three convolutional layers for feature extraction and ReLu as the activation function to improve computational efficiency in the CNN DL method, Zhang et al. [51] obtained the highest accuracy rate in the literature on the GTSRB dataset.

The work of Zhang et al. [151] revealed that using streaming video data rather than images increased the success rate. On the other hand, these authors argued that the choice to use a DNN model should be made after considering the computational complexity, energy consumption, and memory requirements of processing video streams. If there is a need to perform tracking alongside traffic sign detection, it can be useful to employ video-based models.

Among traffic sign recognition studies in the literature that employ a CNN architecture, the network design typically includes two or three convolution layers, which is a common design in image recognition applications. It is observed that max-pooling is preferred in the pooling layer, the kernel size is set to 3 × 3, and the stride value is set to 1 or 2, because traffic sign recognition datasets tend to be relatively small. In studies where traffic sign detection and recognition functions are carried out together, a two-stage approach is often adopted. In the first stage, possible traffic sign regions are detected with R-CNN, after which traffic sign classification is performed by training the DNN for each possible region [152].

Sindhu et al. [153] presented an overview of object recognition methods using CV techniques, applications related to traffic sign detection and recognition, and model and performance evaluations, discussing the advantages and disadvantages of the proposed techniques in detail and the several existing problems which need to be resolved.

Table 4 contains a list of traffic sign detection and recognition studies in the literature that employ DL methods. Some studies proposed both detection and recognition methods, while in others, only traffic sign recognition methods were explored. It can be seen from Table 4 that CNN and its variants are widely used in traffic sign recognition research due to their success in image classification problems. Thanks to DL models, automatic feature extraction can be performed, removing the need for tedious handcrafted feature extraction methods, and the traffic sign recognition accuracy rate was increased to 99.84%. It is recognized that the authors of these studies generally prefer the GTSRB dataset for performance evaluation, and also prefer accuracy and mAP as performance measurement metrics.

### 3.3. Vehicle Detection and Classification

One of the most important components of safe driving is vehicle detection. Detecting vehicles in images or video frames using CV techniques is a widely researched subject in the field of ITS, as these systems can provide useful insights about traffic at much lower costs compared to their traditional sensor-based counterparts. Vehicle detection with CV techniques has many real-world applications, such as automatic license plate recognition, queue prediction, speed detection, traffic jam and accident detection, and the detection of various anomalies. However, due to factors such as camera hardware limitations, camera movement, object occlusion, object speed, object resolution, low traffic density, and the complexity of lighting conditions, vehicle detection remains a challenging problem in the literature [155].

CNN-based DL methods are widely adopted for vehicle recognition tasks. It is noted that the regions in which vehicles are likely to be found are identified using a two-stage approach, followed by verification, and that customizations are made in line with different application needs [152].

For vehicle detection, Zhu et al. [156] and Huang et al. [157] proposed models based on YOLO. In [157], a system that even works at night was developed. In another study [158], a domain-adaptive region-based CNN (Faster R-CNN) was developed for parameter estimation of traffic flows. This model can detect vehicles both in daylight and at night.

In autonomous driving studies, which have gained momentum in recent years, it is critical for vehicles to be able to perceive and analyze their environment in real time. For an autonomous vehicle to proceed safely along a route, it should be able to detect its position relative to other vehicles. CV-based DNN models are widely used for this purpose. Camera systems with different types of sensors are employed to detect and classify objects in the environment [39]. The issue of vehicle recognition in autonomous vehicles has been explored by many researchers, including Lange et al. [39], Du et al. [52], and Wu and Lin [53]. Light detection and ranging (LIDAR) sensors were used in [39] to identify areas containing potential vehicles. In order to detect moving vehicles, a fixed number of tracking points were determined in a certain region in [53], after which the vehicles were detected from the movement clues. In [159], the authors focused on the development of a video analysis processor for the detection and classification of vehicles in urban areas, adopting a fuzzy-set-based approach.

A study on vehicle detection applications in changing environments was presented in [160], categorizing vehicle detection methods into appearance-based and motion-based approaches. Special illumination, weather, and driving scenarios were also explored in terms of methodology and quantitative evaluation where sensor fusion was suggested for effective on-road vehicle detection. A comprehensive review of vehicle detection techniques under varying moving cast-shadow conditions using DL-based CV techniques was conducted in [155], along with a comparative analysis of shadow-detection and -removal algorithms. The authors observed that although state-of-the-art techniques outperformed compared to other approaches in terms of performance, and they are recommended for the removal of shadows, there is a trade-off between accuracy and high processing times.

Another issue in the field of ITS is the extraction of class and definition information, such as the models and colors of vehicles. There are different fields of vehicle classification, including vehicle type recognition (car, motorcycle, truck, etc.), model recognition, and brand recognition. These applications—which are of vital importance, especially for security systems—can enable the identification of vehicles with desired features in the big data obtained from hundreds of traffic camera images. In addition, vehicle classification is actively used in smart transportation systems, as well as fleet tracking and parking systems. Among the models developed for vehicle classification, models developed using DL techniques [54,161,162] occupy a large part of the literature.

Table 5 contains a list of vehicle detection and classification studies in the literature that employ CV methods. As the table shows, DL approaches have achieved good results on vehicle detection and classification tasks in recent studies. Some studies present both vehicle detection and classification methods, while others focus solely on vehicle detection. It can be further observed from Table 5 that CNN-based methods such as YOLO variants and Faster R-CNN are preferred in vehicle detection and classification studies due to the high success rates they have attained in recent works. Through the application of DL models, a recall rate of 97.9% in vehicle detection and an accuracy of 99.03% in vehicle classification were achieved. It is recognized that the authors generally prefer different types of datasets for performance evaluation, and that they prefer to use accuracy and mAP as performance measurement metrics.

### 3.4. Pedestrian Detection

One of the CV applications needed in autonomous driving and video surveillance contexts (for example, optimizing pedestrian waiting times at signalized intersections [165]) is pedestrian detection, which is a specific application of the object recognition problem [166]. Pedestrian detection is one of the most well-established areas of CV study for ITS [19,20,167,168] and is used as a preliminary step to ensure traffic safety and determine pedestrian density.

Much of the early research in this field focused on the detection style framework, in which a sliding window detector is shifted over the image and used to detect people in a frame [169]. Pedestrian/human detection from images is usually performed through monolithic or parts-based recognition. Among the monolithic sensing approaches, the methods proposed in [143,170,171,172] are traditional pedestrian detection methods that typically train a classifier using features extracted from a full body, employing Haar wavelets [173], HOG [143], edgelet [174], and shapelet [175] features. Various learning approaches, such as SVM, boosting [176], and RF [177], have achieved varying degrees of success in pedestrian detection. Although these approaches are successful in low-density crowd scenes, they are adversely affected by high-density crowds. Therefore, researchers have attempted to solve this problem by adopting part-based detection methods [178], which create amplified classifiers for specific body parts such as heads and shoulders.

Another problem encountered in the pedestrian detection context is that of occlusion. Tian et al. [179] divided images into square cells to overcome this problem, classifying each cell as a part of the body (such as a shoulder, arm, or leg); for their part, Zhang et al. [180] proposed a method based on the Faster R-CNN architecture.

A method for estimating the number of pedestrians using perspective-corrected measurements of foreground areas was proposed in [181]. Two parametric approaches (standard linear regression model and linear discriminant analysis) and two nonparametric approaches (probabilistic neural networks and k-nearest neighbors) were evaluated to find the best mapping between area measurements and the number of people in the area. Because this method does not require very large datasets to train the classifier, it is suitable for counting pedestrians in public areas.

CV algorithms for detecting pedestrians in individual monocular images, referred to simply as pedestrian detectors, were the focus of [169], which presented a comprehensive evaluation of pedestrian detection technologies using traditional CV approaches and ML techniques. In this study, a large, well-annotated, and realistic monocular pedestrian detection dataset was created, and the statistics of pedestrian size, location, and congestion models in urban scenes were examined. In addition, a refined per-frame evaluation methodology was proposed that enabled research and informative comparisons to be conducted, including measuring performance on scale and occlusion. Authors evaluated the performance of sixteen pre-trained pedestrian detectors on six datasets.

Many existing works dealing with the pedestrian detection task have focused on crowd analysis. Low-level density methods, which are among the density-based approaches used to model crowds, are mostly based on motion elements obtained from the frame-by-frame modeling for individual object detection. Pedestrian localization methods were proposed in [182,183], while crowd behavior analyses were proposed in [184,185]. In [56], crowd scene analysis was performed on a train station dataset in an attempt to understand and model pedestrian behavior in crowds using a CNN method.

It can be observed that CNN networks are widely used for pedestrian detection. Ouyang and Wang [57], Fukui et al. [58], and John et al. [59] can be considered among the first authors to have studied DL-based pedestrian detection applications.

The use of additional data sources to improve pedestrian detection performance is also an approach adopted by the authors working on this topic [3]. To create a dense depth map, Schlosser et al. [60] used data from a LIDAR sensor, from which three features representing different aspects of the 3D scene were extracted. It was noted by Liu et al. [104] that training thermal images with CNNs provides additional information that can be used to distinguish pedestrian samples. Luo et al. [186] proposed a switchable RBM so as to model visual variations at different levels, as well as to address clutter in the image background and variations in pedestrian appearance. To tackle the multi-scale problem, Li et al. [187] proposed another network structure, called scale-sensitive fast R-CNN. In this study, the authors applied multiple subnets to detect pedestrians in disjoint ranges, then adaptively combined them to produce the final detection results.

A recent review of crowd-counting and density-estimation methods with a particular focus on CNN-based approaches was presented in [188]. In this work, remarkable enhancements obtained using CNN-based methods were compared with hand-crafted representations; the drawbacks of existing CNN-based approaches were also discussed. A comprehensive review of CNN-based methods for crowd behavior analysis was presented in [189], which explored optimization methods used in CNN-based approaches, the fundamental and innovative methodologies employed, and a taxonomy that summarizes important aspects of the CNNs. Focusing on pedestrian detection, abnormal activity detection, and activity detection more generally, in [190], the authors examined the techniques, applications, and datasets used for automatic visual human behavior detection systems covering the period from 2000 to 2014, where SVM- and neural-network-based methods were popular for prediction tasks and progress was required for behavior representation in dynamic scenes and reasoning for interpretation and action. A density-aware pedestrian proposal network (DAPPN) for human detection in crowded scenes was developed by Yun et al. [191]. This study presents two networks, namely a proposition network and a selection network. The algorithm begins with pedestrian detection, then moves on to a crowd density map. This study used a traditional CNN method for feature extraction and carried out tests on the WorldExpo10 and PETS2009 crowd scene datasets.

In [166], the results of research into pedestrian detection using DL methods, occlusion, and multiscale problems affecting pedestrian detection were examined in detail. The authors observed that AI models developed in recent years can successfully detect pedestrians in images with high precision. However, authors emphasized that there is still a lot of room for research to provide real-time performance improvements and lighten the model while ensuring detection accuracy. In addition, each pedestrian can be followed individually through the use of video processing techniques [117]. The article published by Brunetti et. al. [192] reviewed the use of DL-based video processing methods such as CNN, DNN, RBM, and GMM for pedestrian detection. The authors analyzed vision-based pedestrian detection systems based on their field of application, acquisition technology, CV techniques, and classification strategies and discussed the classification performances on various benchmark datasets.

Table 6 presents a list of pedestrian detection studies in the literature using CV methods. It can be observed that the performance of pedestrian detection studies is generally measured in terms of the average miss rate (%) metric, and moreover, that Faster R-CNN and other CNN-based DL methods have recently been used for pedestrian detection tasks. The transformer architecture is notably successful at detecting pedestrians from video images, with an mAP value of 100%. It is also observed that the Caltech, KITTI, and ETH datasets are widely preferred for performance comparison.

### 3.5. Lane Line Detection

The automotive industry has become one of the largest industries in the world. As a result, the detection of roads and lanes has become crucial to the success of ADAS. In light of the risk of lane-departure-related automobile traffic accidents resulting in death or injury, the detection of lane markings and lane departure situations is considered to be an important element of driving assistance systems that can improve road safety, reduce traffic accidents, and prevent loss of life and property damage [194].

Today, CV-based lane line detection methods can be broadly divided into two categories: (i) traditional image processing techniques, and (ii) semantic segmentation methods, including DL techniques. Traditional image processing techniques include feature-based and model-based approaches and can be classified as either similarity- or discontinuity-based. Model-based approaches contain different parametric values and consist of straight line, curve, or pattern models. Semantic segmentation, which is employed in lane departure warning systems (LDWS) research, is among the new research trends, and includes various ML, ANN, and DL methods. Image processing algorithms for lane line detection and semantic segmentation methods (including ML, neural network, and DL methods) used for LDWS were analyzed and compared by Chen et al. [194]. However, the authors highlighted that there is still a lot of work to do for LDWS research and development due to factors such as bad weather, vehicles affecting each other, system action speed, enthusiasm of users, and the alarm system.

Traditional lane line detection essentially comprises five steps: (i) obtaining the image containing the lane line, (ii) determining the lane region, (iii) enhancement of the current region, (iv) feature extraction, and (v) lane line modeling. With traditional CV methods, lane lines are usually detected using methods such as color enhancement, Hough transform, and edge detection [82].

In recent studies, it is observed that DL and ANNs are used to replace manual markings, and a certain number of learning feature detectors have been created to perform lane segmentation at the pixel level. Gopalan et al. [195], who utilized pixel methods, took advantage of pixel-hierarchy feature descriptors to model the contextual information of lane lines and used boosting algorithms to select relevant features during the detection of lane markings. Kim and Lee [61] combined CNN with the random sample consensus (RANSAC) algorithm to detect lane lines and used CNN for image enhancement when the road scene was complex. Presenting an experimental evaluation of DL methods in highway driving, Huval et al. [62] proposed a CNN model capable of selecting and classifying relevant features for lane marking. Li et al. [63] used a multitask deep convolutional network to find geometric lane features such as position and location, along with a recurrent neural network for lane detection. DNNs were employed by Lee et al. [196] for lane and road detection and recognition processes in day and night conditions, with a particular focus on low-lighting and adverse weather conditions. In their research, Dewangan and Sahu [105] developed different semantic segmentation models for the perception of roads, pavements, lanes, and lines using convolutional networks.

DL-based approaches, which offer many advantages compared to traditional image processing techniques, require a training dataset of sufficient size to train the model for accurate and fast lane line detection. Therefore, DL methods should be developed based on multi-sensor data and the advantages of traditional image processing algorithms [194]. DL-based lane detection methods, along with their advantages and limitations, were discussed in [197], while [198] presented an overview of lane detection and tracking algorithms for driver assistance systems, along with the tools/datasets used, performance evaluations, their advantages and disadvantages, problems encountered, patented products, and a SWOT (strengths, weaknesses, opportunities, and threats) analysis of different approaches. Similarly, [199] comprehensively examined research into lane marking with DNNs and DL techniques, explaining their successes and their limitations. Studies reveal that some challenges still remain that need further investigation, such as computational complexity, lack of generalization, and real-time computing in autonomous vehicles and mobile devices.

An overview of existing lane line detection studies is provided in Table 7. As the table shows, the majority of these studies aimed to use DNN frameworks to ensure that vehicles can detect lane lines and stay in their lanes. It can further be observed that datasets such as Caltech, TuSimple, and BDD100K are commonly used for performance evaluation, in addition to datasets produced by the authors. In lane line detection studies employing CV methods, where different performance evaluation metrics (such as F1 score, mIoU, AUC, and accuracy) are used, it is notable that the lane line detection success rate was excellent, reaching an F1 score of 100% up to 50 m.

### 3.6. Obstacle Detection

One of the main functions of smart vehicle navigation systems is the detection of obstacles in transportation infrastructures. It is important for an intelligent vehicle system to be able to detect obstacles, adjust its speed after assessing the position and size of an obstacle, and navigate while considering obstacles. In particular, it can be observed that passive vision systems are thought to be a superior option in future autonomous vehicle scenarios, and that researchers have been interested for some time in obstacle detection works based on camera images alone.

Many existing obstacle detection methods based on CV techniques focus on detecting only certain types of obstacles, such as cars or pedestrians. However, this can result in a significant number of false-positive detection alarms, or in systems missing obstacles that need to be detected. For this reason, different types of sensors that also provide environmental sensing, such as LIDAR sensors, are used in obstacle detection [83].

Recently, [83] presented a literature-mapping study focusing on CV techniques that use cameras for obstacle detection in smart navigation systems. In this study, the authors analyzed approaches based on (i) image segmentation (IS), (ii) stereo vision (SV), (iii) optical flow (OF), and (iv) neural networks. Obstacles were classified according to their characteristics and detection targets as either vehicles, pedestrians, or obstacles in general.

IS is an image transformation and simplification technique that works by dividing the image into parts or segments that can then be analyzed and classified one by one. In the image segmentation process, the properties of pixels (such as color, texture, and density) and their spatial relationships are considered. There are image segmentation methods that take pixel location and similarity into account, as well as image segmentation methods that decompose objects by considering large changes in pixel density levels (discontinuity) [83]. Some of the most commonly used techniques for discontinuity detection are the Sobel filter and Canny edge detection [203]. In [204], the authors used the graph-cut segmentation method to segment vehicles found in images. In [205], grouping was achieved by conducting analysis based on pixel location and similarity to label different objects. A discontinuity-based approach using the Harris operator for edge detection was presented in [206]. In [22], the authors devised a similarity-based approach, using the direct sparse odometry–simultaneous localization and mapping (DSO-SLAM) technique to generate the point cloud and the k-means clustering method to obtain the edge regions.

OF is an image-feature extraction technique for extracting relative movement information from corresponding regions of successive frames in a scene. The technique is based on the idea of representing the displacement of patterns in video frames as a vector field, referred to as the optical flow field [83]. Lucas–Kanade [207] is a traditional OF calculation method which utilizes an image registration method that uses the spatial density gradient of the images to find a good combination. The Gunnar–Farneback algorithm is a more recent method for OF [208]. In this method, which was developed to produce a dense OF approach that works on a point grid, information from two consecutive frames is used to extract the displacement vector information. Among the other studies in the literature that detect obstacles using OF information are [209,210,211].

SV is another image feature extraction method that aims to extract 3D information from image sets obtained simultaneously from different vantage points and calculates depth based on the disparity between these images. SV is the most widely used approach in CV-based obstacle detection studies [83]. In the studies presented in [212,213,214,215,216,217,218,219,220,221], the researchers employed the SV method for obstacle detection. In most of these studies, different techniques have been used for preprocessing and post-processing.

It is evident that ANNs have been widely used in recent CV studies for obstacle detection. For example, in the object detection and classification research presented in [222], an ANN based on fuzzy logic achieved a success rate of 92%. Good results were obtained in most recent studies using CNN architectures, which require little data preprocessing and can process large amounts of data for self-feature extraction. The Mask R-CNN [64], RetinaNet [223], and YOLOv3 [224] models, which are among the newest CNN models used in obstacle detection, were compared by the authors in [225], who found that the Mask R-CNN method achieved higher accuracy than the other two methods. In [226], where the performances of the SVM, YOLO, and single-shot multibox detector (SSD) methods were compared for obstacle detection, it was observed that SVM performed poorly compared to the CNN-based approaches, the YOLO algorithm worked faster, and SSD provided more accurate results.

It can further be observed that only a few of the reviewed studies used a single method for obstacle detection; in many studies, hybrid approaches that utilize a combination of different techniques were preferred. In [227], the IS and SV methods were used together; in [228,229], the ANN and SV methods were used together; in [230], Haar-like features, IS, and principal component analysis with histograms of oriented gradients (PCA-HOG) were used together, while objects were classified with SVM. In [106], the authors used the SV and HOG methods together with the histograms of flow (HoF) technique; [107] employed the OF method of the forward–backward error algorithm; in [231], HOG was used together with cascade classifiers and Haar-like properties; [232] employed global and local features; finally, in [233], the HOG, hypothesis generation, and SVM methods were used together by the authors.

Badrloo et al. [234] reviewed the image-based obstacle detection techniques for the safe navigation of unmanned vehicles and autonomous vehicles. The authors explored two groups of algorithms: (i) monocular algorithms and (ii) stereo-based methods. They concluded that while monocular-based approaches are simple and computationally fast, stereo-based methods are not computationally cost-effective and require a powerful graphics processing unit (GPU). Moreover, the authors observed that despite recent studies focused on DL-based methods for fast and accurate obstacle detection and significant progress in recent years, they still face challenges in complex and unknown environments where there are objects with varying types and shapes.

A list of obstacle detection studies with CV methods is presented in Table 8. The SV, IS, HOG, and OF methods are widely used by researchers in obstacle detection studies; however, the table also shows that recent studies tended to focus on DNNs, and obstacle detection studies are increasingly carried out by employing autoencoder (AE) methods and the YOLO series. Along with the obstacles in the road network, it is noticed that pedestrians and vehicles are also detected in some obstacle detection studies. There are also supporting techniques used in the obstacle recognition studies in the literature, such as (i) the occupancy grid map, which represents a map of the environment with grids, (ii) ROI, where a region of the image in which obstacles are most likely to occur is selected, and (iii) inverse perspective mapping, which performs a geometric transformation that shifts pixels from 2D to 3D and remaps them to a new position in a new inverted 2D planar image [83].

### 3.7. Anomaly Detection in Video Surveillance Cameras

As the number of surveillance cameras in cities continues to increase, an enormous number of video streams are now being recorded every moment. It has become physically impossible to monitor, analyze, and make sense of the content of such videos through human effort. Accordingly, there is a need for systems that can learn from the available normal data to detect unusual events in videos. Unlike the usual video-based action- or event-recognition problems, in which each class is properly identified and labeled, anomaly detection problems are based on learning only the normal data distribution and considering anything that occurs outside this distribution to be an anomaly. For this reason, the video anomaly detection problem can be considered as a one-class problem in which all other classes are unknown [268].

To perform anomaly detection, raw video images collected through cameras are subjected to pre-processing, followed by feature extraction. The obtained data are then passed through a modeling algorithm, in which a learning method models the behavior of surveillance targets and determines whether the behavior is anomalous [8].

Detection methods that can automatically detect anomalies in videos have been in development for more than a decade. The video anomaly detection methods in the extant literature were reviewed in detail in [8]. Anomaly detection methods in video surveillance cameras can be categorized as either (i) learning-based or (ii) model-based. Learning-based algorithms learn anomalies or normal states based on labeled (supervised learning) or unlabeled (unsupervised learning) training data; it is also possible to use semi-supervised learning methods that combine small amounts of labeled data with large amounts of unlabeled data. Model-based approaches include statistics-based, proximity-based, classification-based, reconstruction-based, and prediction-based approaches, as well as methods such as fuzzy theory prediction, the adaptive sparsity model, sparsity-based background extraction, the use of high-frequency correlation sensors, particle filtering, and the redundancy removal approach. Various other techniques are also employed in the literature to detect anomalies in traffic flows [8].

While researchers in earlier studies focused on the use of various handcrafted spatiotemporal features and traditional image-processing methods, more advanced techniques such as ML methods have recently been used for object-level information acquisition and tracking, for classification and clustering, and for the detection of anomalies in video scenes [8].

The USCD [269], UMN [270], and UCF crime datasets [271] are some of the publicly available datasets used in anomaly detection research. However, when using these datasets, it is difficult to determine whether a network needs to focus on learning motion patterns, object interactions, or something else in order to successfully generalize for an anomaly detection system [268].

A brief survey of the contemporary methods developed between 2015 and 2018 for anomaly detection in videos is presented in [268], which classifies these methods according to their network structures and the datasets used. In anomaly detection using video surveillance cameras, DL-based methods have achieved high performance under harsh environmental conditions [272,273,274,275,276]. DNNs with hierarchical feature representation learning are much more powerful than the handcrafted feature extraction techniques used in traditional architectures [95].

In [277], the authors proposed to cascade 3D DNNs in order to detect and localize anomalies. First, a motion threshold was applied to grid points (in frames over time), such that only significant and moving grid points were retained for the next stage. A classification module was then applied to these remaining points to determine whether anomalies were present. In [272], a CNN + LSTM-based network was adopted to detect anomalies in the UCSD [269] and Subway [278] datasets. In a relatively similar network proposed in [279], a Convolution3D-based approach incorporating LSTM was used to extract landmarks from videos. These extracted landmarks were then used to check whether anomalies were present, assuming that the videos contained anomalies [268].

An AE with SVM was used by Tran and Hogg [280], AEs with convolutional LSTM were used by Ryan and Savakis [87], and a stacked RNN framework was used by [274]. Temporally coherent sparse coding was proposed as an effective anomaly detection method for datasets in [269,278,281]. A self-learning supervised learning method using Convolution3D was proposed by [276]. An interesting use of GAN for anomaly detection was proposed by [282].

In [88], a convolutional LSTM (ConvLSTM) network in an encoder–decoder model was proposed for anomaly detection for future frame prediction and reconstruction. The same architecture was proven to be a promising method for video anomaly detection in [89]. Input video frames were sent to a convolutional LSTM network for feature extraction, then reconstructed using deconvolution. Luo et al. [274] proposed a temporally coherent sparse coding (TSC) approach in which similar neighboring frames were mapped to the reconstruction coefficient via stacked RNNs. In [90,283], the authors used stacked convolutional LSTM networks in an AE architecture for feature extraction in video sequence data.

Stacked AEs were used in [284,285,286] to learn the distinctive features of appearance, motion, and their common representations, which were classified by SVM to find anomalous events. Authors utilized CNN for feature extraction in [65,66,67]. Following recent advances in ML, several studies have experimented with the use of CNNs, Conv3D, LSTMs, and similar architectures in the field of video anomaly detection [268].

Fuzzy theory estimation, adaptive sparsity models, sparsity-based background extraction, use of high-frequency correlation sensors, particle filtering, and redundancy removal are among the other methods used in the literature for the detection of anomalies in traffic flows, such as accidents, unsafe driving behavior, road crimes, and traffic violations [8].

The research prepared by Nayak et al. [287] shows the progress made in video anomaly detection using DL techniques. This study presents several DL techniques used in video processing to detect anomalies, such as abnormal activities (fights, riots, traffic violations, stampedes, and unusual objects), weapons, and abandoned luggage. Despite the progress in DL-based methods for video anomaly detection, the authors demonstrated that there still exist several research challenges such as the need for better datasets, reduction in computational complexity, solving incompleteness of the methodology, finding the best evaluation methodologies, the need for co-designing of hardware and software, trade-offs between accuracy and processing time, and the need to address the environmental challenges. In [8], Patrikar and Parate performed a detailed study of the evolution of anomaly detection methods in video surveillance systems, the methodologies used in video anomaly detection, evaluation parameters, datasets and video anomaly detection methods on edge devices, challenges, and future research areas.

Table 9 lists the different methods developed using the Avenue [281], Ped1 [269] and Ped2 [269] datasets, which are among the public datasets most widely used in the literature on anomaly detection in video images using CV techniques. The AUC values used for frame-level performance measurement of these developed methods are also presented in Table 9, expressed as a percentage. As can be seen from the table, DL-based AI techniques such as GAN, LSTM, ConvLSTM, spatial-temporal AE, ConvAE, and VAE are widely used in current literature studies to perform anomaly detection in video surveillance cameras. In the literature, the highest obtained AUC values were 89.82% (with the SVD-GAN method on the Avenue dataset), 98.5% (with the DSTN method on the Ped1 dataset), and 99.21% (with the MLAD method on the Ped2 dataset).

In video anomaly systems where the AUC performance metric is widely used, accuracy and mAP are among the other performance evaluation metrics used by researchers to measure the performance of the developed methods.

### 3.8. Structural Damage Detection

Natural disasters such as floods and earthquakes can cause cracks to appear in important urban infrastructure, such as roads, bridges, and buildings. Millions of dollars are spent each year to detect these cracks. In order to prevent damaged infrastructures from collapsing and transportation infrastructures from being destroyed, and to ensure the functionality and physical integrity of these infrastructures, maintenance processes are usually planned by visually inspecting and assessing the condition of cracks. However, the detection and manual visual inspection of cracks is a very laborious task, as checking them regularly requires a significant amount of human resources. There is therefore a need for the effective and efficient automatic detection of damage to transportation infrastructures [303]. In light of this, the use of CV methods to detect cracks in or damage to transportation infrastructures has become an interesting topic for researchers.

In CV applications, cracks are considered to be abrupt changes in pixel intensity, appearing as thin dark lines on the surface of a solid material where it has been separated without fragmentation. Cracks are mainly classified as (i) fatigue cracks, (ii) block cracks, (iii) edge cracks, (iv) longitudinal cracks, (v) transverse cracks, or (vi) reflection cracks [84]. There are many techniques presented in the literature for detecting these cracks and their depths using image processing methods. While some of these techniques utilize traditional image processing and ML methods, recent studies mainly use models based on CNN architectures, which have yielded improved results compared to more traditional approaches.

It is known that previous studies have achieved good results using a range of traditional image processing techniques for crack detection. Cracks in images can be detected using various techniques, such as edge information [304], morphological processing [305], statistical methods, digital image correlation [306], and model mapping [307]. The crack detection process consists of the following phases: (i) image capture, (ii) image processing, (iii) crack feature extraction, and (iv) crack identification. In crack detection, crack pixels in the image are assumed to be darker (with higher intensity) compared to their neighboring pixels, and crack and non-crack regions are classified by comparing the contrasting information between neighboring pixels [308]. Statistical methods involving threshold values are employed to distinguish between cracked and non-cracked regions in the image [309]. Furthermore, different intensity values are used to determine the probability of pixels in the image being classified as cracked or non-cracked [310].

In [311], a crack-detection and -classification method was developed that reduces the noise in the image and reveals the cracks. Cracked and non-cracked images were classified in [312] using an SVM method incorporating a histogram technique. Classification was performed using binary tree and backpropagation methods, which divided the image into cracked and non-cracked regions by comparing grayscale values.

Otsu’s [313] method helps researchers to perform image segmentation. The PCA algorithm, which is used in the dimension reduction literature, has also been employed to identify cracks in images [314]. Filters were additionally used to detect cracks by combining binary versions of the crack image. In [315], the original image was convolved using filters applied in different orientations [316]. Background pixels were separated from foreground pixels using thresholds, and noise in the image was removed with a Sobel filter. Otsu’s method was then used to detect cracks [316]. The MATLAB-based CrackIT toolbox was proposed in [317] for crack detection. Cracks in the concrete structure were detected by first converting the image to grayscale, then applying the Sobel filter.

Many environmental conditions (including shadows, dust, spot noise, multicolored spots, uneven light illumination levels, multiple background scenes, and changes in the dataset) can make it difficult to detect cracks in an image using traditional image processing methods. To address these challenges, ML-based methods have been utilized to facilitate more successful feature extraction and segmentation [84]. In [318], background objects were removed using ML-based segmentation methods, after which cracks were classified with the SVM method by extracting color and texture features. The ML methods used in the literature for crack segmentation, classification, and detection purposes include DBN [33], simple classifier-based road crack detection and characterization [319], the Markov-based method [320], image binarization [321], RF [322], RNN-based crack detection on a 3D asphalt surface [69], and AdaBoost textural pattern recognition [323].

Although feature-extraction-based ML techniques can perform well on images with clear and visible cracks, they remain insufficient for crack detection in unclear images. On the other hand, DL-based methods have achieved better results compared to traditional image-processing-based methods and other ML-based methods. Cracks can be detected by DL methods via classification, localization, or segmentation. Classification is used to classify images as either cracked or non-cracked, while pixel segmentation is used to classify individual pixels as either cracked or non-cracked [84].

Models based on CNN architectures have been the focus of researchers’ attention in the field of crack detection. Crack detection approaches using CNN architectures can be divided into two groups, namely (i) sliding window and (ii) pixel-level crack detection [84]. An up-to-date, comprehensive analysis and review of CNN-based crack detection models in civil structures can be found in [84]. This study addresses a range of topics, including image preprocessing techniques, software tools, hardware, datasets, CNN network architectures, learning procedures, loss functions, and network performance. The review focuses on the classification and segmentation of crack images using CNN architectures, along with the studies carried out to detect structural cracks.

An automatic road-crack detection method, which classifies input images by learning their distinguishing features, was developed in [70] to promote safe driving. A CNN-based classifier was proposed in [71] to classify damage in steel structures. In [324], a PCA-based approach was used to classify cracks, and a transfer learning method was used to detect cracks from datasets. In [325], the authors proposed a CNN-based model for structural crack detection, while a CNN-based model for crack detection on tunnel surfaces was also proposed in [72]. In [326], a robot-based crack inspection technique was developed to minimize human errors and reduce costs. A DL-based AlexNet DCNN architecture was compared with classical algorithms (including the Roberts, Prewitt, Sobel, Laplacian of Gaussian, Butterworth, and Gaussian algorithms) in [327]. DeepCrack [328], a DL model with encoder–decoder architecture for learning high-level crack properties, was proposed as an end-to-end, trainable, and automatic crack detection method.

The YOLO and YOLOv2 [329] CNN architectures were utilized by the authors in [73] for road crack detection using bounding boxes of appropriate width and height. GoogLeNet [74] was used for crack detection in [75], while the VGG-16 network was used for crack detection in [76]; in this work, a combination of CNN (VGG-16) and RNN was employed to classify cracks as either mild or severe damage.

The genetic algorithm (GA) was adopted to find the optimal values of parameters, such as the number of convolutional layers, kernel size, and the number of kernels in each layer, to build the optimal CNN model for crack detection in [330]. The GA was able to optimize the network depth, the hyperparameters, and the size of the layers, thereby increasing the accuracy of crack detection.

A deep FCN for semantic segmentation designed to perform crack detection and density assessments on concrete crack images was proposed in [331]. CrackSegNet, which is a deep fully convolutional neural network, was proposed in [332] for pixel-based crack segmentation in tunnels. The network consisted of an encoder, a decoder, dilated convolutions, spatial pyramid max pooling, and skip connections, while the backbone network of the encoder path was a modified version of the VGG-16 architecture.

An automatic crack detection method for separating cracks from noisy, illuminated, and textured surfaces, and which uses U-Net-based pixel-level crack classification, was proposed in [333]. Another U-Net-based end-to-end semantic segmentation network for pixel-level crack classification was proposed in [334]. In CrackNet-V [335], which is a pixel-level crack detection method, individual pixels identified in a certain region on a 3D asphalt pavement image were classified as either cracks or non-cracks. In [336], which supports the use of RGB or grayscale images of any size as inputs, a pixel-based deep semantic segmentation network was proposed. An end-to-end encoder–decoder-based DL model for pavement crack detection at the pixel level was proposed in [337]. In [338], which uses a post-processing technique to remove the detected crack distortion and to measure crack width and orientation, a convolutional encoder–decoder network (CedNet) was utilized to segment the cracked pixels.

Using a ResNeXt-based framework, which is a modified version of the original ResNeXt architecture that combines the VGG architecture and the Inception network, the authors in [339] detected cracks in bridge concrete structures. In [340], which is a dense-dilation fully convolutional neural network, a crack detection method for high-resolution images was proposed. A DL semantic-segmentation-based crack detection method was proposed in [341]; in this approach, Mask R-CNN was used to train the crack dataset in an attempt to overcome image processing difficulties caused by factors such as shadows and dirt in the images. A sample segmentation network called APLCNet was proposed in [342] for pavement crack detection.

CNN networks were used by Zhang et al. [69] for pavement crack detection, by Cha et al. [111] for crack detection on concrete surfaces, and by Zhang et al. [70] for road damage detection. Nguyen et al. [77] further proposed a two-stage CNN model for road defect detection.

In [343], the authors proposed an SDDNet architecture for real-time crack segmentation. This method aims to remove background and crack-like features by segmenting the cracks in real time. ARF-Crack, a rotation-invariant fully convolutional network, was proposed in [344]. Adopting the DeepCrack network for crack segmentation, active rotating filters were used to encode the rotation-invariant property into the network.

In [112], Kortman et. al. investigated the shortcomings of road damage detection algorithms that meet the requirements of autonomous driving systems, exploring the architecture of environmental sensing systems and existing road damage detection algorithm designs. The authors proposed two lightweight DNNs, one for road damage detection and the other for damage severity detection, as central components of the system.

State-of-the-art systems and algorithms for road imaging and pothole detection were investigated by Ma et al. [345]. In this research, (i) classical 2D image processing, (ii) 3D point cloud modeling and segmentation, and (3) ML/DL methods for pothole detection were analyzed in detail. The current challenges and future development trends of CV-based pothole detection approaches were also discussed, and it was argued that classical 2D-image-processing-based and 3D point cloud modeling and segmentation-based approaches were becoming obsolete, having serious limitations. These authors further argued that CNNs have achieved success in pothole detection, and moreover that self/unsupervised learning for multimodal semantic segmentation seems to be a promising avenue for future research.

The literature shows that CNN-based crack classification and segmentation methods outperform traditional image processing techniques and other ML methods. It was further observed that although the sliding window technique can effectively classify cracks, it is not efficient enough for localizing crack pixels and segmentation. Encoder–decoder architectures such as U-Net, SegNet, and FCN architectures yield highly efficient crack segmentation results. Moreover, the objective function plays an important role in minimizing the errors, and the selection of the appropriate loss function contributes significantly to network performance [84].

Image-processing-based and ML-based crack detection methods were presented in [303], which provided an in-depth discussion of the methods used in crack detection, datasets, application areas, performance results, features used, and limitations of the methods in the existing literature. Authors showed that CNN is the most frequently used technique for crack detection and that most of the recent studies focused on using ML and DL methods instead of image processing techniques. In [346], the authors investigated different image processing techniques for crack detection in engineering structures. This study discussed various image processing techniques, including (i) camera-based, (ii) infrared (IR)-based, (iii) ultrasonic-image-based, (iv) laser-image-based, (v) time of flight diffraction (TOFD)-based, and (vi) other methods, but it lacks the state-of-the-art techniques that utilize ML- and DL-based approaches. Gaps in the literature and problems encountered were also presented.

A list of existing studies on structural damage and defect detection is presented in Table 10. As the table shows, DL methods, which are variants of CNN architectures, are predominantly preferred in recent structural damage detection studies that utilize CV techniques. Methods of this kind have obtained detection accuracy values that reach up to 99.39%. It can further be observed that CV methods have been utilized for different purposes, such as crack detection in bridges, crack detection in noisy concrete surfaces, crack detection in pavement, crack detection in roads, road defect detection, and structural damage detection. While the accuracy metric is commonly used to measure the performance of these proposed methods, metrics such as AUC, AIU, F1 score, recall, precision AP, and mIoU were also used for the performance evaluations.

### 3.9. Autonomous Vehicle Applications

Autonomous vehicle systems, which are among the most innovative forms of ITS, have the potential to provide a range of economic, environmental, and social benefits to society by delivering a customized user experience, improving traffic management, increasing road network capacity, and making roads safer for users. Especially in the automotive sector, the adoption of data-driven AI and ML models has opened up new horizons in new services and business models, such as autonomous fleet management, driverless trucks, and robotaxis [361].

Autonomous vehicles that can detect obstacles and accurately read traffic signals by combining CV and robotics technologies will be among the key applications in the future that will rely heavily on DL models.

Obstacle detection, scene recognition, and lane recognition are among the prominent problems needing to be solved in the autonomous vehicle context [80]. In order for autonomous vehicles to continuously capture and analyze the surrounding environment in real time, they need to use DL techniques, along with other sensor technologies; that is, they need to learn the semantic meaning of traffic-related information. Autonomous vehicles rely on messages from external sources to perform actions critical to driving safety and increase efficiency in an environment consisting of both static (vehicles parked on the roadside, buildings, trees, etc.) and dynamic objects (pedestrians, road signs, lane markings, traffic lights, etc.). To position itself in a dynamic environment, the autonomous vehicle needs to perceive its surroundings and create a map of this environment; to achieve this, it will need to continuously capture and analyze its surroundings in real time, using systems such as cameras, LIDAR, radar sensors, and roadside infrastructure.

Data transmission and processing are among the basic functions of autonomous vehicles [4]. AI technologies, which also include CV techniques, play a role in processing and making sense of these data, improving the driving safety of autonomous vehicles, reducing traffic accidents, and increasing driving and traffic safety more broadly. The data collected by the vehicle, along with CV techniques and other ML methods, are used to adjust the physical controls of the vehicle (steering, acceleration, and braking) and provide the ability to plan and make appropriate decisions autonomously [361]. The sensing systems of autonomous vehicles need to accurately detect non-static objects and predict their behavior, as well as detect static objects and recognize the information they convey [362].

In [81], the authors developed an end-to-end learning method for autonomous vehicles using CNNs. In [80], where two controllers were used simultaneously, CNN networks were utilized to obtain the appropriate steering angle in order to keep the autonomous robot in the lane. Bojarski et al. [79] trained a CNN to map raw pixels to steering commands. These authors developed an end-to-end learning approach for autonomous vehicles that takes the raw image as an input and automatically generates the control signal. Compared to planning autonomous driving by individually addressing problems such as lane marking detection, path planning, and controlling, end-to-end learning methods were able to optimize all process steps simultaneously. Another end-to-end learning approach was proposed by Chen and Huang [82] to obtain the appropriate steering angle to keep an autonomous vehicle in its lane. The CNN model developed by these authors took raw image frames as inputs and determined the steering angles accordingly. The model was trained and validated using the comma.ai dataset, which consists of front-view image frames and steering angle data captured while driving on the road. After end-to-end model training was complete, the proposed method could steer the autonomous vehicle directly using the front-view camera data.

A sequential end-to-end transfer learning method was proposed in [363] to estimate left and right ego-lanes directly and separately without any post-processing. It was shown by Maqueda et al. [364] that, using ResNet, the vehicle steering angle for autonomous vehicles could be accurately predicted under a wide range of conditions.

Chen et al. [86] proposed a new model for autonomous vehicles, called the brain-inspired cognitive model with attention. The proposed model is comprised of three parts: (i) a CNN to simulate the human visual cortex, (ii) a cognitive map describing the relationships between objects in a complex traffic scene, and (iii) an RNN, which is combined with a cognitive map updated in real time to implement the attention mechanism and LSTM.

In [13], Vishal et al. proposed a real-time traffic light recognition method for autonomous vehicles by blending the traditional ML and DL methods together through a visual sensor. In this study, YOLO was used for traffic light detection, while the SVM method was used to classify the states of traffic lights.

In the autonomous driving context, scene understanding, contextual information extraction, and decision making using sensor data all play a crucial role. In [114], the authors analyzed the research area of scene understanding, which is mostly based on computationally complex DL models.

Mahaur et al. [365] presented a study on the detection of road objects (vehicles, pedestrians, traffic signs, traffic lights, etc.) using DL-based algorithms. This study carried out a detailed and systematic comparative analysis of five DL-based road object detection algorithms (R-FCN, Mask R-CNN, SSD, RetinaNet, and YOLOv4) on the large-scale Berkeley Deep-Drive (BDD100K) dataset. Experimental results were calculated using the mAP metric and inference time. By precisely calculating various practical metrics such as model size, computational complexity, and the energy efficiency of DL-based models, the study provides researchers with a comparative evaluation of the results of popular DL-based object detection algorithms for road target detection in autonomous driving applications.

Galvao et al. [362] presented a review of autonomous vehicle perception systems, specifically those designed for pedestrian and vehicle detection. This study noted that while both traditional and DL techniques were used for pedestrian and vehicle detection, DL techniques produced the best results, and a combination of different detection algorithms were shown to improve accuracy performance. Despite good detection rates achieved, the authors argued that current methods still encounter challenges to detect small, occluded, and truncated objects. It is emphasized that there is still further research needed under bad illumination and weather conditions using challenging datasets.

Estimating the correct distance between an autonomous vehicle and the objects in its trajectory is vital if the vehicle is to move safely through its environment. Parrotta et al. [366] presented a proposal to estimate this distance in a real-world scenario through an on-board camera, with the support of a rover, arm platforms, and sensors. The proposal includes the use of an interpolation technique to estimate the distance with good accuracy.

Table 11 lists some of the CV studies in the literature on autonomous vehicle/robot applications (it should be noted here that studies on traffic sign detection and recognition, pedestrian detection, lane recognition, obstacle recognition, etc., were being conducted by researchers before the advent of autonomous vehicle research). As can be seen from the table, various types of CNN architectures are utilized for different purposes in autonomous vehicle/robot applications. CV techniques are also used for various purposes, such as safe and robust navigation to a specific destination in any environment, object (vehicle, pedestrian, cyclist, etc.) recognition, determining the appropriate steering angle to keep the vehicle in its lane, estimating left and right ego-lanes, detecting and recognizing traffic lights, classifying pedestrian traffic lights, detecting free spaces and boundaries for existing and adjacent lanes, estimating distances to obstacles and vehicle behaviors, obstacle detection, and target tracking. Both the datasets and performance criteria employed can be observed to vary depending on the type of application. In autonomous vehicle/robot applications where CV methods are employed, it is evident that solutions with varying success rates have been developed for detection, recognition, and prediction in different studies.

### 3.10. Other Applications

It can be observed that CNN networks have been widely utilized in CV studies with the goal of significantly reducing human intervention and lowering operating costs [3] in several types of ITS-relevant applications. For example, Xue and Li [375] and Makantasis et al. [376] used CNN networks in tunnel inspection activities, Ramos et al. [377] used them to detect minor road hazards for autonomous vehicles, and Chen et al. [78] used them to inspect catenary support devices for defects.

The problem of non-recurring congestion caused by accidents, road construction works, or special events was studied in [378]. The authors proposed and described DxNAT, a DNN for non-recurring congestion prediction. In the study, traffic data were paired with images, and a CNN was applied as a classifier.

In order to highlight image regions, an attention model was applied by Kim and Canny [93] to visually mark decision cues. In [379], the authors utilized DL methods for real-time parking lot detection. Pan et al. [380] explored utilizing traffic cameras to detect snow and ice on the road in winter conditions.

CV methods have also replaced manual security checks at subway stations in China. In Shanghai, an identity verification system powered by facial recognition technology was introduced at train stations. Passengers can pay for their ticket and be granted entry into the station by scanning their faces. The system automatically compares the passengers’ information with the photo on their ID card and makes a match [381].

Automatic traffic accident detection is another important emerging issue in traffic monitoring systems. Today, many signalized intersections are equipped with surveillance cameras connected to traffic management systems. CV techniques offer many suitable tools for automatic accident detection. One such framework for accident detection at intersections for traffic surveillance applications was presented in [38]. The proposed method consists of three hierarchical steps, including (i) efficient and accurate object detection based on the state-of-the-art YOLOv4 method, (ii) object tracking based on the KF, combined with the Hungarian algorithm for association, and (iii) accident detection via trajectory conflict analysis. Vehicle–vehicle, vehicle–bicycle, and vehicle–pedestrian collisions, along with other potential accidents occurring at the intersection, could be detected by the proposed method.

An examination of the literature reveals that CV methods are utilized in many ITS applications, including vehicle counting, vehicle speed detection, average traffic speed detection, lane departure warning [233], driver/vehicle tracking, video-based toll collection, speed enforcement, and parking violation detection. A study of CV applications designed to improve safety, operational efficiency, security, and the enforcement of laws in road transportation systems was presented in [7]. In [382], the authors examined ML methods and publicly available datasets that model the direction of a driver’s gaze by analyzing the driver’s spatiotemporal viewpoints for driving assistance and automation applications. Moreover, the authors provided a summary of current challenges and open issues, such as the availability and quality of data, evaluation techniques, and the limited scope of attention modeling, that need to be solved to make attention-based driving assistive systems applicable in automated systems.

Since the detection of driver drowsiness and fatigue is the most effective way to prevent a large proportion of sleep-related traffic accidents, a real-time drowsiness detection system (RT-DDS) was proposed in [383], which can be applied in motor vehicles with the help of traditional CV applications. AI technologies can also help with law enforcement, such as by detecting people who are driving drunk or texting while driving [4].

A technique for detecting, recognizing, and tracking pedestrians, vehicles, and cyclists along a tram route in a complex urban environment was presented in [384]. The proposed method utilized CV and DL approaches and the YOLOv3 algorithm. The research results showed that the proposed method can very accurately investigate and detect the location and speed of road users near and on the rails in front of the tram.

In [385], Sathyanarayana addressed various methods used for vehicle detection and classification, focusing on CV- and DNN-based techniques, with an emphasis on electronic toll collection. The advantages and disadvantages of the various approaches were also discussed.

Table 12 presents a list of other applications in the literature where CV techniques are used in the field of ITS. As can be seen from the table, CV techniques are used for various purposes in an ITS context. It can be observed that DL-based CV methods are actively used in many areas: from fully automatic tunnel inspection to the detection of concrete defects in tunnels; from red light signal duration detection using low-resolution CCTV cameras to minor road hazard detection; from non-recurring traffic jam predictions to the detection of non-recurring traffic anomalies caused by specific incidents; from the automatic intelligent classification and detection of tunnel surfacing defects to the optimization of signal phases; from automatic traffic volume analysis at road junctions to drowsiness and fatigue detection; from parking occupancy detection to vehicle counting and vehicle queue length estimation; from real-time accident detection using traffic cameras to snow and ice detection, etc. It can further be observed that the metrics used to measure performance vary based on the type of application being developed, and that, especially in recent years, CNN-variant DL techniques have achieved excellent performance across a range of tasks and evaluation metrics.

## 4. Discussions and Perspectives

After reviewing the WoS, ACM, and IEEE databases, this survey has identified and analysed over 300 studies pertaining to CV applications in the field of ITS, along with the techniques employed, datasets utilized, areas of development explored, and potential impacts of CV studies on ITS observed in the surveyed research. Notably, however, the CV applications encountered in the field of ITS extend beyond what is presented in this survey. The following sections present a summary of the applicability of CV applications in ITS, as well as their contributions, challenges, shortcomings, and potential future avenues for development.

### 4.1. Applicability

Looking at the increasing significance of CV studies based on DL methods in the field of autonomous and connected mobility, we foresee that the use of CV applications in real-time ITS will increase in the future. We think that DL-based CV techniques could be used to efficiently solve the complex problems encountered in intelligent transportation, provided that sufficient training data are available or can be produced at low cost [3]. On the other hand, an increase in the use of CV applications in ITS would raise concerns about potential violations of individual rights. For example, the development of facial recognition technologies for use in transportation systems could lead to concerns that governments might impose more oppressive policies on individuals; this in turn raises the possibility that the use of facial recognition systems in the transportation sector could be banned. Therefore, we anticipate that in the coming years, CV applications that do not violate personal rights will be widely used to increase the level of intelligence and safety of transportation systems, as well as to make the transportation infrastructure more accessible, especially for disadvantaged groups and vulnerable road users.

### 4.2. Contributions of Computer Vision Studies

It is predicted that, through the effective application of CV and AI methods, the efficiency of transportation systems can be increased, resulting in numerous economic gains. For example, through the use of CV methods, daily losses in city traffic management and automated parking systems can be prevented. CV-based solutions can play an active role in alleviating traffic congestion, minimizing excessive fuel consumption, saving fuel and time, and reducing carbon emissions.

Thanks to CV and AI solutions, road infrastructure and signaling systems can be adaptively shaped to distribute traffic more homogeneously by anticipating future demand. It is estimated that an efficient AI-based traffic management system can reduce waiting times at signaled intersections by up to 47% and ensure constant traffic flows at optimum speeds [395]. Traffic forecasting will enable road users to select the most time- and energy-efficient routes, leading to reductions in emissions, fuel consumption, air pollution, and noise pollution; non-exhaust emissions will also be reduced, since a smoother flow of traffic will lead to less braking overall.

The development of autonomous transportation systems, especially those in which CV techniques play a critical role, has the potential to reduce travel times and road maintenance costs. It has been reported that fully autonomous transportation systems will generate cost savings across Europe totalling 38 billion Euros [395]. It is also expected that accident rates will decrease significantly with the spread of autonomous vehicles; this will also reduce damage to public property and healthcare costs incurred due to injuries. According to another statistic, it is estimated that 1–2% of the USA’s general health budget could be saved once the use of autonomous vehicles becomes widespread [396].

Another economic impact of CV–ITS interaction is related to reduced energy consumption. Some studies show that, for various deployment scenarios, the use of intelligent cars will result in significant energy savings [397]. The study in [395] reported that affordable travel with AI-enabled vehicles will contribute positively to the environment in various respects, including reductions in air and noise pollution, greenhouse gas emissions, and fuel consumption.

The adoption of CV and AI technologies in the logistics sector is expected to result in the automation of the supply chain, which will increase efficiency and thus reduce costs to a significant extent. In addition, leading companies are declaring that driverless freight deliveries will be a reality in the near future [398]. Self-driving trucks are expected to reduce costs by 40%, as they can travel longer distances without stopping and thus speed up trade [399].

Another contribution in terms of energy is related to fleet tracking systems. It has been reported that convoys of loaded vehicles traveling in a platoon formation will reduce logistics costs; fuel savings of 4% can be achieved through the use of such driving protocols, as the wind resistance will be reduced [174]. Another economic contribution of this system is that these convoys can remain constantly mobile, since driverless vehicles do not need to stop to allow the drivers to rest.

The autonomy that CV and AI techniques will provide is expected to result in further significant benefits to our everyday lives and society as a whole. For example, it is estimated that driverless vehicles will adhere strictly to traffic rules, obey speed limits, and drive without making mistakes, which will reduce accident rates and significantly increase traffic safety. A reduction in the psychological problems caused by traffic congestion and an increase in quality of life are among the indirect advantages that will be gained thanks to the ability to automatically select alternative routes according to traffic conditions and optimize waiting times at signalized intersections. Thus, it is predicted that these AI-supported applications will improve the quality of transportation services in both urban and rural areas, as well as provide a better travel experience in general [395]. This autonomy is further expected to significantly improve the mobility of groups with limited mobility, such as the elderly and the disabled, and expand socialization opportunities for disadvantaged groups.

With the development and widespread use of CV methods, the security aspect of transportation systems is also expected to improve. For example, through the use of image processing technologies built into cameras, it will be possible to instantly detect violent incidents or unusual situations occurring on public transportation or in public areas. Vehicles or people wanted by law enforcement officers will be easily detected, and the routes followed by these people can be inferred by processing millions of data points using DL-based image processing methods.

It is also expected that AI solutions, including CV methods, will birth new sectors and open up new employment opportunities. In particular, the implementation of connected and autonomous vehicle technologies is projected to create 25,000 new jobs by 2035 [395]. In general, this wave of automation in both road transport and supply chain management is likely to optimize the interaction between humans and technology, as well as to open up new jobs and research avenues in the fields of engineering and software development.

Based on the surveyed literature, Table 13 summarizes some of the contributions of CV studies to ITS in terms of economic, environmental, and social aspects.

### 4.3. Open Challenges in Computer Vision Studies

In CV studies in the field of ITS, images obtained from cameras are processed and converted into meaningful information that can be used for different purposes. However, many adverse weather conditions—such as heavy snowfall, rainfall, fog covering the camera, excessive brightness caused by sunlight, and differences in image angles due to camera shaking caused by strong wind—can cause difficulties in obtaining and properly processing the image. If sequential images cannot be obtained stably from camera streams, it is difficult to make meaningful inferences using CV techniques. In addition, other factors such as complex backgrounds and low contrast can also make it difficult to automatically extract features from images.

Although successful results can be obtained from CV applications in ITS, especially those using DL techniques, they also have limitations in terms of the data and computational resources available. The performance of the training environment can be increased by using GPUs [3], which are composed of thousands of parallel processing units and can achieve much higher processing speeds compared to CPUs. However, real-time processing in ITS applications, such as passenger recognition at airports and metro stations, requires cluster setups consisting of large numbers of machines equipped with GPUs and CPUs, the cost of which is relatively higher than those equipped with CPUs alone. These modeling processes also have a significant carbon footprint, meaning that their environmental impact should be taken into consideration.

In DL-based studies, there are many factors that can significantly affect training time: the settings of hyper-parameters (including the learning rate, mini-batch size, number of layers, and number of hidden units in each layer, among others), the choice of activation function, the choice of normalization method, the type of network selected, the hardware used, etc. For this reason, DL studies should be carried out while paying attention to the finer details of these issues and the recommendations presented in relevant studies. In addition, one of the main challenges of these methods is the lack of suitable datasets for use in developing DL models [3].

With the spread of autonomous and connected vehicles, which are expected to play a role in making transportation systems safer and more efficient, the processing power is expected to shift to the mobile chips in the vehicle, which will be the client. This will require more effective DL methods and neural networks to be developed and integrated into these chips [3].

**Figure 5:**
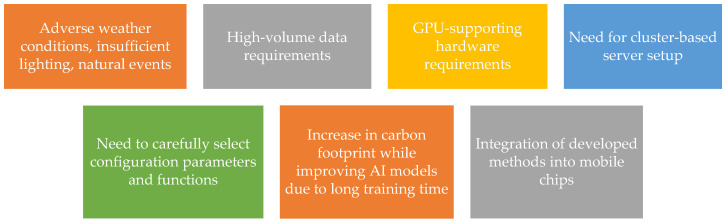
Challenges of Computer Vision Studies.

For more detailed information on DL methods for improving transportation systems, researchers can refer to [3]. The challenges faced in DL-based CV studies are summarized in Figure 5.

### 4.4. Future Research Directions and Trends

Our examination of CV studies in the field of ITS revealed many future research areas and emerging trends. First, more research into real-time traffic sign detection and recognition will be required, due to the critical importance of these processes for autonomous vehicles, and increasing accuracy rates are also an important research area [3].

The use of the attention mechanism [116,180], which assigns different weights to regions in an image in DL models, is among the topics that could be investigated in more depth in the future. This will make it possible for the computers to focus on important areas of the images (which can be subsequently verified as important by humans) [3].

Since many abnormal events occur in real-world traffic scenes, the development of efficient cognitive models to deal with these situations may be another attractive topic for future research [86]. In addition, training models for DL-based methods is a resource-intensive and computationally costly task; accordingly, rather than training the model from scratch, it would be valuable to explore the use of transfer learning approaches, which are based on the idea of using existing trained models. In particular, the use of transfer learning and fine-tuning techniques for the YOLO model used in recent studies on object recognition problems could be explored to produce more accurate results than previous trained models [155]. Other interesting topics for researchers could include training the AI model on real-time systems with lower computational costs and developing methods with less complexity [188]. Furthermore, in light of the studies in the literature, another active exploration area is that of improving the hardware constraints and model training processes in order to spread CV studies in ITS and establish development environments more easily [3].

More research is needed to make automatic license plate recognition algorithms run in different kinds of environments with various non-standardized license plate datasets, to train real-time object detectors such as YOLO for automatic license plate recognition, to detect the license plates of vehicles traveling at high speeds, to evaluate the performances of these systems under conditions of low contrast or insufficient/excessive light, and to test them in real-time scenarios [123].

The performance of image-based lane detection and LDWS could still be improved. Specifically, topics meriting further investigation include determining which factors have the most significant impact on the reliability of lane-detection and lane-departure warning systems, then developing solutions that can adapt to complex geometric road design models, adverse weather conditions, and low-illumination environments. Moreover, systems that can detect lane lines in real time at high speeds with high accuracy while minimizing false alarms also need further exploration. Additionally, methods such as lane-departure detection with multi-sensor fusion and 3D detection algorithms that increase the reliability of lane detection could be examined in more detail in this context [194].

Although many CNN-based DL methods have been proposed for obstacle detection in the existing research, there is still more work to be done on this subject. Challenges such as low-quality road scenarios need to be addressed, given that the vast majority of studies have been conducted using high-quality road images, which may not reflect the real-world situation in developing countries [83].

Considering the interactions between drivers and pedestrians in the ITS environment, one of the main factors needing to be considered in the ITS context is vehicle users. Given that driver faults are one of the most common causes of traffic accidents, driving style plays an important role in ITS, especially for improving driving safety and developing advanced driver assistance systems [400]. In this context, it would be useful to investigate user-oriented detection tasks related to driving style and pedestrian detection in the field of CV.

Many articles in the literature focus only on detecting cracks in transportation infrastructures, but most of these studies do not calculate crack sizes. Measurements such as the length, width, density, and depth of the detected cracks provide important clues about the condition and durability of the component and can help the transportation units make decisions regarding the subsequent use of the structure. For this reason, more focus should be placed on the measurement and classification of cracks in transportation infrastructures. The image-processing-based system to be developed should be able to support the decision mechanisms of transportation authorities regarding the type and status of the cracks detected using different methods, along with the steps to be taken. In addition, more research is needed to eliminate noise and other irregularities in images, detect structural cracks (especially segmentation at the pixel level), and address unbalanced datasets [84,303].

One of the disruptive effects of CV studies in the transportation context will be in the logistics sector. It is expected that solutions such as truck convoys and truck systems with autonomous driving capabilities will reduce costs and increase productivity in the sector. However, there is a need to intensify research in this field in the areas of environmental factors, time, and fuel savings, since CV studies will be effective in transforming the logistics industry.

Although good detection results have been obtained for pedestrians and vehicles in autonomous vehicle systems, current algorithms still have difficulty detecting small, closed, and truncated objects. Further research is needed in this area, as there are limited studies on how to improve sensing performance in challenging light and weather conditions [362].

Ice on the road, manhole defects, floods, and potholes are among the factors that negatively affect driving safety. Since these problems can be detected with CV algorithms and then quickly solved by teams trained for this purpose, it will be beneficial to conduct research into models that will enable road and driving safety units to work in close cooperation with researchers.

Traffic camera systems enable vehicle flows to be monitored in real time, and the streams of data flowing continuously from thousands of cameras quickly become a huge information stack. Using video analysis tools in the field of CV, processing raw data with AI methods, and edge computing will make significant contributions to ITS. AI models, which can be integrated quickly into existing systems, will be able to produce efficient reports on matters such as traffic density information, average speed, and accident detection through camera stream data. In addition, these systems will be able to assist security forces in finding wanted persons or vehicles.

In light of the information presented in Chapter 3, we expect that future research will focus on CV applications based on DL methods that can facilitate the performance of complex functions in the field of autonomous driving. We believe that autonomous driving architectures will come to dominate in the future, revolutionizing transportation systems and transforming ITS, and that the development of CV techniques will play a critical role in this sector. Despite the advances in CV research related to autonomous and connected vehicle technologies, there are still areas, however, that need to be improved. Moreover, it is anticipated that there will also be a need to develop real-time video anomaly detection, automatic accident detection, and real-time crowd analysis [189] frameworks, although there is comparatively less research on these topics in the existing literature.

Since DL-based CV techniques require large amounts of data to produce good results, a more detailed review of the literature on data collection, big data processing, and strategies for generating value from data should be conducted. In addition, there is a need for open-access datasets that will facilitate the development of academic studies in the field of CV. A data-based governance approach to transportation systems should also be established by organizing workshops with experts in the public and private sectors. In this regard, it would be beneficial to develop initiatives that create a synergy between academia and public- and private-sector organizations.

GAN can be used to create fake videos of specific people or produce evidence of events that never occurred. It is therefore possible that GANs could be used maliciously to create images and videos that constitute a risk to the reputations, or even personal safety, of individuals. Accordingly, future research should focus on improving fraud detection and processes to efficiently and effectively detect AI-generated images, including those developed using GANs [401].

**Figure 6:**
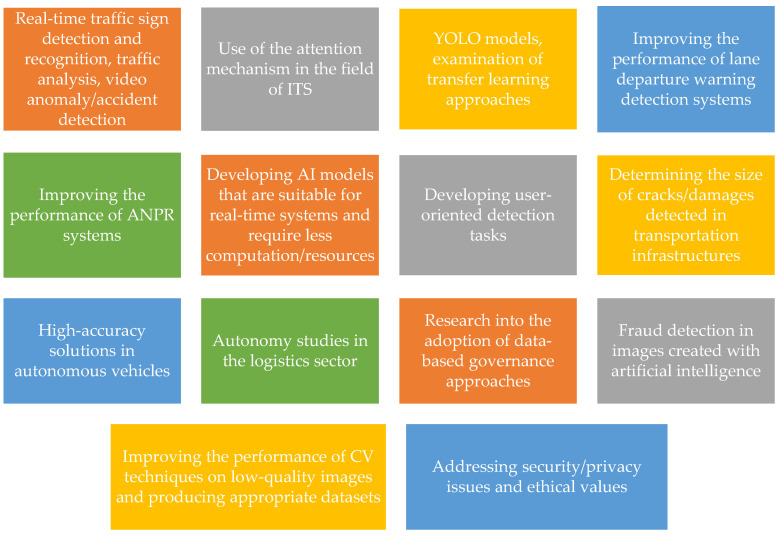
Future Research Areas and Trends.

In addition, if CV-based solutions are to be adopted by and spread throughout the public and private sectors, any related security and privacy issues need to be addressed and handled meticulously. For this reason, future researchers should focus on developing approaches that will enable CV applications to be used without raising concerns about security, the vulnerability of institutions, or ethical issues associated with the use of AI technologies. Future areas of study for researchers and global trends regarding CV research in the field of ITS are summarized in Figure 6.

## 5. Conclusions

Intelligent transportation systems—which can be defined as integrated transportation management systems consisting of advanced data communication, information processing, and traffic management technologies—can instantly process and analyze real-time data collected from heterogeneous sources to facilitate better decision making [3]. ITSs, being among the most important components of smart cities, aim to improve efficiency, mobility, traffic safety, and the environmentally friendly and sustainable development of transportation systems [1]. As complex interconnected systems that connect vehicles, traffic lights, drivers, sensors, roadside units, and other infrastructure, ITSs offer many innovative applications, such as optimal traffic signal control, safe intersection crossing, and emergency alert notifications; these systems can also enhance travel efficiency, increase public safety, improve emergency response procedures, and significantly improve citizens’ quality of life [402].

This survey comprehensively discusses the usage areas of CV applications in the field of ITS, the technologies employed, the contributions of CV techniques, the difficulties and limitations encountered, and future research areas and trends. In particular, the evolution of CV studies from past to present in the field of ITS—such as automatic license plate recognition, traffic sign detection and recognition, vehicle detection and classification, pedestrian detection, lane line detection, obstacle detection, anomaly detection in video surveillance cameras, structural damage detection, and autonomous vehicle applications— is analyzed in detail, and the results of the relevant studies are presented. After evaluating more than 300 studies, it can be concluded that CV technologies have many applications in increasing the intelligence level of ITSs and supporting the construction of safer and more efficient transportation systems.

From the review of the literature, it can be observed that a shift has occurred from traditional ML methods to DL-based approaches in many recent intelligent transportation applications (such as automatic license plate detection, traffic sign detection and recognition, vehicle and passenger tracking, obstacle detection, lane line detection, video-based surveillance, and structural damage detection applications). It has also been recognized that CNN-based architectures are widely used, especially for handling CV problems, and that DL methods can be considered the most effective choice available [3], as they provide remarkable performance advantages. With the development of ML, DL, and big data analytics methods, along with the availability of more powerful computational resources, CV applications will be used extensively in ITS contexts. Thus, traffic congestion and negative effects on the environment caused by transportation will be reduced; moreover, these systems will contribute to efficient and safe traffic management and increase the air quality in cities. In other words, the development of technology will facilitate the establishment of a greater and deeper connection between CV techniques and transportation systems, along with the transformation of transportation systems into smarter ones. It will also provide insights to relevant institutions and organizations that will support the automatic performance of various tasks in the transportation industry, as well as helping the relevant parties to make quick decisions in an emergency, determine what additional features should be included to make transportation systems safer, and identify areas where there is potential for further research and investment [4]. Thanks to advanced ML algorithms, it will be possible to quickly detect and resolve irregular situations in traffic; the detection of criminal behavior will also be accelerated.

Since it would not be possible to cover all existing literature on CV applications in the field of ITS, a representative subset containing examples of current approaches has been selected for detailed analysis and review in this survey. In addition, since a detailed analysis and evaluation of all methods used in ITS-related CV studies would be beyond the scope of this study, references are provided in each CV application section for review articles that can be examined for more information.

Furthermore, it has been observed that most of the DL models were developed in Python or MATLAB environments. Python’s PyTorch, Tensorflow, Caffe, and Keras frameworks and MATLAB’s Computer Vision Toolbox are widely used in CV studies in the field of ITS. It has also emerged that the performance of the developed algorithms was evaluated not only on the datasets that were most widely used in the relevant application areas but also on datasets produced by the study authors. This survey has further highlighted a need for new datasets that can be used to evaluate system performance in challenging conditions, such as adverse weather, poor lighting, complex backgrounds, or low-quality images.

In summary, CV techniques, which have groundbreaking potential in computer science, will bring significant new functions to transportation systems as DL methods continue to develop. These techniques will increase the intelligence level of transportation systems and will become dominant in future research. We anticipate that CV techniques will offer several opportunities for developing countries and will be effective in improving the autonomy of transportation systems. We hope that this study will serve as a basis and a reference for the advancement of CV research in the field of ITS, for the enhancement of the capabilities and performance of transportation systems, and for identifying promising new research areas in ITS.

## Figures and Tables

**Table 1:** Problems Addressed by CV Techniques in the Field of ITS.

Computer Vision Function	Application Areas	Sample Datasets	Performance Metrics
Object Detection	Problem: Boxing the objects in the image/video and finding their coordinates in the image Detection of traffic lights Detection and classification of traffic signs Pedestrian detection Detection of vehicle type and vehicle counting	COCO CityScape ImageNet LISA GTSDB (German Traffic Light Detection) Pascal VOC CIFAR-10/CIFAR-100	mAP (mean average precision) Accuracy Precision Recall AP (average precision) RMSE (root mean squared error)
Object Segmentation	Problem: Classifying the pixels of the objects in the image and thus obtaining the individual masks of the object Speed estimation Determination and tracking of road lines Route optimization	COCO CityScape BD100K KITTI LISA	mAP
Image Enhancement	Problem: Restoring images that have been corrupted by low lighting, haze, rain, and fog Removal of raindrops on images obtained from camera sensors Bringing low-resolution objects up to high resolution Sharpening blurry images Conversion of fish-eye camera systems to Cartesian coordinate system	REDS	PSNR (peak signal-to-noise ratio)
Object Tracking	Problem: Tracking objects in video Tracking of pedestrians and vehicles Vehicle speed detection Route extraction	MOT19	MOTA (multiple object tracking accuracy)
Event Identification/Prediction	Problem: Making sense of what happened in the video Accident recognition/prediction Congestion estimation Detection of dangerous situations and routes	UCF101 Kinetics600	Accuracy mAP
Anomaly Detection	Problem: Detection of abnormal behavior in transportation systems Pedestrians/objects suddenly appearing on the road Anomalies that may arise in rail systems Detection of improper driver behaviors (drowsy/drunk driving, text messaging, cell phone use, etc.) Detection of traffic rule violations and suspicious vehicles with automatic license plate recognition systems	UCSD Ped1 UCSD Ped 2 Avenue UMN UCF Real World Street Scene CIFAR-10/CIFAR-100 ShanghaiTech	AUC (area under curve) Accuracy mAP
Density Analysis	Problem: Determining the density of pedestrians, passengers, or vehicles Density analysis in public transport contexts Automatic detection of traffic jams Determination of vehicle density in parking lots Detecting the density of pedestrians in certain locations	Oxford 5K UCSD Mall UCF_CC_50 ShanghaiTech WorldExpo’10	MAE (mean absolute error) MSE (mean squared error)
Image/Event Search	Problem: Extraction of certain vehicles, pedestrians, or license plates from existing visual archives Searching of target plates in traffic camera archives for law enforcement units Searching digital archives for people or vehicles for security purposes Detection of similar objects belonging to a certain object category.	Oxford 5K Pascal VOC	Accuracy

**Table 2:** ANPR Studies with CV Methods.

Ref. ^1^	Year	Author(s)	Method	Dataset	Recognition Rate
[129]	2015	Ahmad et al.	OCR1: Template matching OCR2: PNN	141 images	OCR1 for E1: 81.99% E2: 78.65% E3: 81.50% OCR2 for E1: 82.42% E2: 78.36% E3: 77.95%
[125]	2016	Hommos et al.	OCR algorithms	958 images	99.50%
[130]	2017	Omran and Jarallah	Back- propagation neural network (BPNN)	60 images	93.2%
[126]	2017	Farhat et al.	OCR using field- programmable gate array (FPGA) processing unit	454+ images, 2790 characters	99.50%
[128]	2017	Sasi et al.	ANN	Blurred, night, and daylight license plate images	N/A ^2^
[40]	2018	Laroca et al.	Data augmentation, distant CNNs for letters and digits	SSIG dataset: 2000 frames; UFPR-ALPR: 4500 frames	SSIG: 97.83% UFPR-ALPR: 90.37%
[127]	2018	Molina-Moreno et al.	Scale-adaptive model, empirically constrained deformation model	2600+ images, multiple datasets (OS, Stills&Caltech, AOLP)	98.98%
[135]	2018	Desai and Bartakke	Tesseract’s OCR	1300 images	92.12%
[138]	2019	Singh et al.	Region of interest (ROI)- based filtering, vertical edge detection with removal of long edges	1000+ videos	92.31%
[139]	2019	Sferle and Moisi	OCR; template matching	110 images	N/A
[140]	2019	Slimani et al.	Template matching	Set 1: 533 Set 2: 651 Set 3: 757 Set 4: 611 (video sequences)	Set 1: 98.1% Set 2: 96.37% Set 3: 93.07% Set 4: 92.52%
[41]	2019	Hashmi et al.	RT-ALPR (CNN)	4800 car images	85%
[21]	2020	Pustokhina et al.	Optimal k-means with CNN (OKM-CNN)	FZU Cars, Stanford Cars, and HumAIn 2019 Challenge Dataset	mAP values: FZU Cars: 96.3% Stanford Cars: 94.8% HumAIn 2019 Challenge Dataset: 96.1% Overall acc: 98.1%
[132]	2020	Silva and Jung	YOLO-based CNN	SSIG, UFPR, OpenALPR	SSIG: 89.15% UFPR: 65.62% OpenALPR: 85.19%
[85]	2020	Gong et al.	Convolutional RNN (CRNN), Deep CNN (DCNN), RNN, spatial transformer networks (STN), and connectionist temporal classification (CTC) models	Chinese City Parking Dataset (CCPD)	93.56%
[133]	2020	Akhtar and Ali	Random Forest classifier	350 images of Croatian vehicles	90.9%
[100]	2020	Darapaneni et al.	YOLOv3, HAAR Cascade, and OpenCV	300+ images; tested on 20+ car images	YOLOv3: 100% HAAR Cascade: 57.8% Open CV: 35.7%
[134]	2020	Calitz and Hill	The design science research methodology	34 vehicles for each angle	96%
[101]	2022	Vetriselvi et al.	DL-VLPNR (Tesseract OCR, Faster R-CNN + Inception V2)	FZU Cars and HumAIn 2019	98.6%

^1^ Ref. refers to References, ^2^ refers to Not Available.

**Table 3:** Traffic Sign Recognition Studies with Traditional ML Methods.

Ref.	Year	Authors	Method	Dataset	Accuracy
[146]	2011	Rajesh et al.	Simple neural network	GTSRB	94.73%
[147]	2011	Boi and Gagliardini	SVM	GTSRB	96.89%
[142]	2012	Zaklouta et al.	k-d trees and random forests	GTSRB	97.2%

**Table 4:** Traffic Sign Detection and Recognition Studies with DL Methods.

Ref.	Year	Authors	Method	Detection	Recognition	Dataset	Accuracy
[42]	2011	Ciresan et al.	CNN	✖	✔	GTSRB	99.15%
[43]	2011	Sermanet and LeCun	CNN	✖	✔	GTSRB	99.17%
[44]	2012	Ciresan et al.	CNN	✖	✔	GTSRB	99.46%
[45]	2014	Jin et al.	CNN	✖	✔	GTSRB	99.65%
[46]	2015	Haloi	CNN	✖	✔	GTSRB	99.81%
[47]	2015	Qian et al.	CNN	✔	✔	GTSRB + MNIST + CASIA	99.83%
[48]	2016	Changzhen et al.	CNN	✔	✔	Chinese traffic sign dataset	99%
[18]	2016	Li and Yang	RBM-CAA, SVM	✖	✔	GTSRB	96.68%
[25]	2016	Li et al.	RBM-CAA, R-CNN, cuda-convnet	✔	✔	LISA-TS (US traffic signs)	96.68%
[49]	2016	Jung et al.	CNN	✔	✔	Korean traffic signs	N/A
[50]	2017	Zeng et al.	CNN	✖	✔	GTSRB	99.54%
[51]	2017	Zhang et al.	CNN	✖	✔	GTSRB	99.84%
[103]	2022	Xing et al.	Faster R-CNN + improved YOLOv5	✔	✔	GTSDB, FRIDA database	95.30% (mAP for Faster R-CNN net), 95.63% (accuracy for improved YOLOv5)
[154]	2022	Marques et al.	YOLOv3 and YOLOv3_tiny	✔	✔	RoboCup Portuguese Open Autonomous Driving Competition; also tested on public roads	Competition YOLOv3: 99.08% (mAP) YOLOv3_tiny: 98.47% (mAP) Public Roads YOLOv3: 98.914% (mAP) YOLOv3_tiny: 95.584% (mAP)

**Table 5:** Vehicle Detection and Classification Studies with CV Methods.

Ref.	Year	Authors	Method	Detection	Classification	Dataset	Performance
[39]	2016	Lange et al.	Caffe CNN	✔	✖	MadeInGermany	~80% (precision)
[52]	2017	Du et al.	PC-CNN	✔	✖	KITTI	89.4% (AP)
[53]	2018	Wu and Lin	OF + CNN (CaffeNet)	✔	✖	7587 images	97.9% (recall)
[159]	2018	Neto et al.	Fuzzy-set-based approach	✔	✔	Different cameras in different scenarios	Ranging between 89.3–100%
[11]	2018	Mittal et al.	Faster R-CNN, SVM	✔	✔	IITM-HeTra	88.7% (AP): Two-wheelers; 98.6% (AP): light motor vehicles; 90.5% (AP): heavy motor vehicles
[55]	2019	Shvai et al.	Ensemble classifiers: CNNs + Gradient boosting-based classifier	✔	✔	VINCI Autoroutes French network	99.03% (Classification accuracy)
[156]	2020	Zhu et al.	MME-YOLO	✔	✖	Roadside Dataset	91.63% (mAP)
[163]	2020	Wong et al.	CNN	✔	✔		93.8% (accuracy)
[157]	2021	Huang et al.	M-YOLO (Mobilenet v2 + YOLO v3)	✔	✖	5576 nighttime traffic scene pictures	94.96% (AP)
[158]	2021	Li et al.	Region-based CNN, Faster R-CNN	✔	✖	2200 traffic images	89.66% (mAP, Night-4)
[54]	2021	Pillai et al.	Deep CNN	✔	✔	Vehicle type: TAU Vehicle Type Recognition Competition Dataset, CompCars Vehicle color: 15,601 vehicle images with eight color classes	89% (vehicle classification accuracy), 95% (color classification accuracy)
[162]	2021	Niroomand et al.	SSFCM (Semi-Supervised Fuzzy C-Mean)	✖	✔	Swiss Motor Vehicle Information System, Federal Office Technical Information, Vehicles Expert Partner	84.78% (avg. accuracy)
[164]	2022	Jiaoand Wang	YOLOv5, KF	✔	✖	Cooper Dr. and N. Lamar Blvd. Traffic images from Austin, Texas, USA	RMSE: 10 (KF), 40 (IoU-based algorithm)
[12]	2022	Alam et al.	Gentle adaptive boosting algorithm + Haar-like features, HOG + SVM	✔	✖	3000 images	97% (AP for daytime), 94% (AP for nighttime)

**Table 6:** Pedestrian Detection Studies with CV Methods.

Ref.	Year	Authors	Method	Dataset	Performance
[167]	1990	Ali et al.	Moving objects detectors (MODS)	Image data acquisition with a CCD camera	N/A
[19]	1997	Oren et al.	Wavelet template, bootstrapping, SVM	Database of frontal and rear images of people in outdoor and indoor scenes	Detection rate: 69.7% (81.6%)
[20]	1998	Papageorgiou and Poggio	Overcomplete dictionary of Haar wavelets and SVM	Image data acquisition with digital image cameras and a digital video camera	Detection rate: > 80%
[168]	2000	Zhao et al.	Stereo-based segmentation and neural network	Urban street scenes	Detection rate: 85.4%
[57]	2013	Ouyang and Wang	CNN	Caltech and ETH	Avg. miss rate computed from AUC (%): ETH: 34% Caltech: 30%
[186]	2014	Luo et al.	Switchable restricted Boltzmann machine (SRBM)	Caltech, ETH	Log-average miss rate: Caltech: 37.87% ETH: 40.63%
[58]	2015	Fukui et al.	CNN-based random dropout and ensemble inference network (EIN)	Caltech, Daimler Mono Pedestrian Benchmark Dataset	Miss rate: Caltech: 37.77% Daimler Mono Pedestrian Benchmark: 31.34%
[59]	2015	John et al.	Adaptive fuzzy c-means clustering and CNN	LSI	Log-average miss rate: 34%
[179]	2015	Tian et al.	CNN (DeepParts)	Caltech	Miss rate: 11.89%
[60]	2016	Schlosser et al.	CNN	KITTI	9.3% improvement in best threshold on KITTI Hard subset
[104]	2016	Liu et al.	Faster R-CNN, Multispectral DNN	KAIST	Miss rate: 36.99%
[193]	2017	Du et al.	Fused deep neural network (F-DNN)	Caltech	Log-average miss rate for “All” setting: 50.55%
[180]	2018	Zhang et al.	Faster R-CNN	CityPersons, Caltech, ETH	Log-average miss rate based on “Reasonable + Heavy occlusion (R + HO)” metric: CityPersons: 41.45% Caltech: 20.03% ETH: 35.64%
[187]	2018	Li et al.	Scale-aware fast R-CNN (SAF R-CNN)	Caltech, INRIA, ETH, KITTI	Log-average miss rate: Caltech: 9.32% INRIA: 8.04% ETH: 34.64% AP on KITTI Hard subset: 60.42%
[117]	2022	Zang et al.	Multi-direction and multi-scale Pyramid in Transformer (PiT)	MARS and iLIDS-VID	Cumulative matching characteristic (CMC) curve and mAP MARS CMC (Rank-10): 98.04% mAP: 86.80% iLIDS-VID CMC (Rank-10): 99.80% mAP: 100.0%

**Table 7:** Lane Line Detection Studies with CV Methods.

Ref.	Year	Authors	Method	Dataset	Performance
[195]	2012	Gopalan et al.	Pixel- level feature descriptors, robust boosting algorithm	Visual inputs from a camera mounted in front of a vehicle	Accuracy in terms of the position error of detected lane markings: 5 × 5-pixel neighborhood, 93.5%
[61]	2014	Kim and Lee	CNN + RANSAC	Complex video clips	Corrected detection rate: Case 1: 94.7.0% Case 2: 93.9% Case 3: 93.2%
[62]	2015	Huval et al.	CNN	Highway dataset consisting of 17K image frames	F1 score: 100% up to 50 m
[63]	2017	Li et al.	Multitask deep CNN + RNN	Video clips (own dataset), Caltech dataset	AUC values: Own dataset: RNN: 99% – Caltech dataset: RNN: Set 1: 99%, Set 2: 93%, Set 3: 96%, Set 4: 99%
[196]	2017	Lee et al.	Vanishing point guided network (VPGNet)	20000 images with 17 lane and road marking classes (own dataset), Caltech dataset	F1 score values: Own dataset: Scenario 1: 87%; Scenario 2: 78.8%; Scenario 3: 76.8%; Scenario 4: 74.3% Caltech dataset Set 1: 88.4%; Set 2: 86.9%
[200]	2018	Wang et al.	LaneNet: lane edge proposal + lane line localization	Real-world traffic data; more than 5000 annotated front-view images taken on both highways and urban roads	True positive rate (TPR): 97.9% False positive rate (FPR): 2.7%
[201]	2019	Hou et al.	Self-attention distillation (SAD)	TuSimple, CULane, and BDD100K	Accuracy for TuSimple: ResNet-18-SAD: 96.02% ResNet-34-SAD: 96.24% ENet-SAD: 96.64% – Accuracy for BDD100K: ResNet-18-SAD: 31.10% ResNet-34-SAD: 32.68% ENet-SAD: 36.56% – F1 Score for CULane (Category Normal): ResNet-18-SAD: 89.8% ResNet-34-SAD: 89.9% ENet-SAD: 90.1%
[202]	2019	Van Gansbeke et al.	Generating coordinate weight map + a differentiable least-squares fitting module	TuSimple	Accuracy: 95.80%
[105]	2021	Dewangan and Sahu	U-Net, Seg-Net, fully convolutional network (FCN)	Camvid	Mean intersection over union (mIoU) value: U-Net: 94%; Seg-Net: 92%; FCN: 86%
[26]	2022	Liu et al.	Reinforced attention method (RAM)	CULane, TuSimple	Accuracy for CULane: 90.80% Accuracy for TuSimple: 96.26%

**Table 8:** Obstacle Detection Studies with CV Methods.

Ref.	Year	Authors	Method	Supporting Methods	Detection Category	Other Features
[235]	2007	Gavrila and Munder	SV and IS	ROI	Pedestrians	–
[209]	2007	Shen et al.	OF	ROI	Obstacles	–
[236]	2007	Kubota et al.	SV	–	Obstacles	Results at night and in the rain
[237]	2007	Ma et al.	IS	Inverse perspective mapping	Pedestrians	Results in foggy and rainy weather
[221]	2008	Franke et al.	SV and OF	Occupancy grid map	Obstacles	–
[238]	2008	Cabani et al.	SV and IS	–	Obstacles	–
[239]	2008	Suganuma et al.	SV	–	Obstacles	Vehicle recognition in a tunnel
[240]	2009	Keller et al.	SV	ROI	Pedestrians	–
[241]	2009	Chiu et al.	SV and HOG	–	Vehicles	Results at night and on rainy days
[242]	2009	Ess et al.	SV and IS	Occupancy grid map	Pedestrians	–
[243]	2009	Ma et al.	IS	ROI, occupancy grid map	Pedestrians	–
[231]	2010	Hota et al.	HOG, cascade classifiers, Haar-like features	–	Vehicles	–
[106]	2010	Walk et al.	SV, HOG, HoF	–	Pedestrians	–
[244]	2010	Li et al.	SV	–	Obstacles	–
[108]	2010	Pantilie and Nedevschi	SV and OF	–	Obstacles	–
[245]	2011	Baig et al.	SV	ROI	Vehicles	Vehicle recognition in a tunnel
[246]	2011	Nieto et al.	IS	ROI	Vehicles	Vehicle recognition in a tunnel
[247]	2011	Na et al.	SV	–	Vehicles	–
[248]	2012	Iwata and Saneyoshi	SV	–	Obstacles	–
[249]	2012	Boroujeni et al.	IS and OF	–	Obstacles	–
[250]	2012	Lefebvre and Ambellouis	SV and IS	–	Vehicles	–
[107]	2013	Liu et al.	Forward–Backward error algorithm and OF	–	Obstacles	–
[251]	2013	Trif et al.	SV and IS	–	Vehicles	–
[252]	2013	Khalid et al.	SV and IS	ROI	Vehicles	–
[253]	2014	Petrovai et al.	SV and IS	ROI	Obstacles	–
[254]	2014	Iloie et al.	SV and HOG	ROI	Pedestrians	–
[255]	2015	Poddar et al.	IS	–	Obstacles	–
[256]	2015	Jia et al.	OF	–	Obstacles	–
[218]	2015	Benacer et al.	SV	–	Obstacles	–
[257]	2016	Wu et al.	SV and IS	ROI	Obstacles	–
[258]	2016	Carrillo and Sutherland	SV and IS	ROI	Obstacles	–
[224]	2016	Redmon et al.	YOLOv3	–	Obstacles	–
[259]	2017	Häne et al.	SV	Occupancy grid map	Obstacles	Cameras around the vehicle
[260]	2017	Prabhakar et al.	Neural network	–	Obstacles	Some results in rainy weather
[64]	2017	He et al.	Mask R-CNN	–	Obstacles	–
[109]	2018	Dairi et al.	SV, deep stacked autoencoder (AE), k-nearest neighbors	–	Obstacles	–
[228]	2018	Dairi et al.	Neural network, SV	One-class SVM	Obstacles	–
[261]	2018	Li et al.	Neural network	–	Obstacles	–
[262]	2019	Fan et al.	Neural network	–	Obstacles	–
[229]	2019	Lian et al.	Neural network, SV	–	Obstacles	–
[206]	2019	Zebbara et al.	IS	–	Vehicles	–
[263]	2019	Hsieh et al.	Neural network	–	Obstacles	–
[264]	2020	Ohgushi et al.	AE with semantic segmentation	–	Obstacles	–
[265]	2021	He et al.	FE-YOLO	Attention mechanism, Downsample-Block, spatial pyramid pooling (SPP) module, CRBlock	Obstacles	Rail crossing obstacle detection
[110]	2022	Ci et al.	DeepLabV3, open-set recognition algorithm	Bayesian probabilistic fusion	Obstacles	–
[266]	2022	Luo et al.	SV	V-disparity image, U-disparity, Stixel method, RANSAC, dynamic programming (DP) algorithm	Obstacles	Obstacle prediction, real-time obstacle detection
[267]	2022	Du et al.	Wasserstein loss-based YOLO model	–	Obstacles	Real-time traffic obstacle detection and classification, different weather conditions, different urban environmental conditions

**Table 9:** Video Anomaly Detection Methods with CV Methods.

Ref.	Year	Authors	Method	Datasets		
				CUHK Avenue [281]	UCSD Ped1 [269]	UCSD Ped2 [269]
[288]	2015	Yan et al.	Two-stream R-ConvVAE	79.6%	75.0%	91.7%
[289]	2016	Hasan et al.	ConvAE	70.2%	81.0%	90.0%
[290]	2016	Colque et al.	Histogram of optical flow (HOF)	N/A	72.7%	87.5%
[272]	2017	Chong and Tay	ST-AE	80.3%	89.9%	87.4%
[89]	2017	Lu et al.	ConvLSTM-AE	77.0%	75.5%	88.1%
[291]	2017	Zhao et al.	3D-ConvAE	80.9%	92.3%	91.2%
[292]	2018	Lee et al.	STAN	87.2%	82.1%	96.5%
[293]	2018	Kiran et al.	CovnLSTM-AE	84%	74%	81%
[273]	2018	Liu et al.	Flownet + U-Net	85.1%	83.1%	95.4%
[102]	2019	Duman and Erdem	OF-ConvAE-LSTM	89.5%	92.4%	92.9%
[91]	2019	Li et al.	U-Net, ConvLSTM	84.5%	83.8%	96.5%
[294]	2019	Zhou et al.	AnomalyNet	86.1%	83.5%	94.9%
[96]	2019	Song et al.	GAN	89.2%	90.5%	90.7%
[295]	2019	Vu et al.	Multi-level anomaly detector (MLAD)	52.82%	82.34%	99.21%
[296]	2020	Chen et al.	U-Net	87.8%	89%	96.6%
[297]	2020	Nawaratne et al.	Incremental spatiotemporal learner (ISTL)	76.8%	75.2%	91.1%
[298]	2020	Sun et al.	Adversarial 3D AE	88.9%	90.2%	91.1%
[299]	2020	Bansod and Nandedkar	Histogram of magnitude and momentum (HoMM)	N/A	82.31%	94.16%
[97]	2020	Ganokratanaa et al.	Deep spatiotemporal translation network (DSTN) based on GAN and edge wrapping (EW)	87.9%	98.5%	95.5%
[300]	2020	Song et al.	Ada-Net (adversarial attention-based AE)	89.2%	90.4%	90.3%
[95]	2021	Jackson and Cuzzolin	Singular-value decomposition GAN (SVD-GAN)	89.82 %	73.26%	76.98%
[301]	2021	Li et al.	Spatial-temporal cascade autoencoder (ST-CaAE)	83.5%	90.5%	92.9%
[98]	2021	Chen et al.	Noise-modulated GAN (NM-GAN)	88.6%	90.7%	96.3%
[68]	2022	Sabih and Vishwakarma	CNN + bidirectional LSTM (Bi-LSTM)	N/A	94.8%	96.5%
[92]	2022	Wang et al.	Double-flow convolutional LSTM variational autoencoder (DF-ConvLSTM-VAE)	87.2%	88.4%	88.8%
[302]	2022	Le and Kim	Attention-based residual autoencoder	86.7%	N/A	97.4%
[99]	2022	Huang et al.	Self-supervised attentive GAN (SSAGAN)	88.8%	92.1%	97.6%
[94]	2022	Wang et al.	ROADMAP (multipath ConvGRU-based frame prediction network)	88.3%	83.4%	96.3%

**Table 10:** Structural Damage Detection Studies with CV Methods.

Ref.	Year	Authors	Method	Application	Performance
[347]	1993	Shan et al.	STRUM, SVM, Adaboost, Ran	Crack detection in bridges	95% (accuracy)
[348]	2010	Ying et al.	Median filter, Hessian Matrix, probabilistic relaxation	Crack detection on noisy concrete surfaces	99.03% (AUC)
[310]	2012	Zou et al.	Recursive tree edge pruning	Pavement crack detection	85% (F-measure)
[349]	2012	Landstrom and Thurley	Feature pyramid and hierarchical boosting network (FPHBN)	Pavement crack detection	8.1% (average intersection over union; AIU)
[70]	2016	Zhang et al.	Deep CNN	Road crack detection	86.86% (precision), 92.51% (recall), 89.65% (F1 score)
[23]	2016	Shan et al.	K-means clustering, Gaussian models	Road crack detection	97% (F-measure)
[69]	2017	Zhang et al.	CrackNet (based on CNN)	Pavement crack detection	90.13% (precision), 87.63% (recall), 88.86% (F-measure)
[111]	2017	Cha et al.	CNN + sliding window technique	Detection of cracks in concrete and routing surfaces	97% (accuracy)
[325]	2017	Cha and Choi	Deep CNN (DCNN)	Crack detection	99.09% (accuracy)
[327]	2018	Dorafshan et al.	CNN (AlexNet)	Crack detection	98% (accuracy)
[75]	2018	Li and Zhao	CNN (GoogLeNet)	Crack detection	99.39% (accuracy)
[350]	2018	Zhang et al.	Canny edge detector, dilate operators, Frangi filter	Crack detection in bridges	98.7% (accuracy)
[351]	2018	Yang et al.	CNN (VGG-19)	Crack detection	97.96% (accuracy)
[352]	2019	Kim et al.	CNN + SURF	Crack detection	99.46% (accuracy)
[331]	2019	Dung and Anh	DCNN	Crack detection	98.47% (accuracy)
[353]	2019	Bang et al.	ResNet-152	Crack detection	59.65% (accuracy)
[354]	2019	Hoang et al.	Transfer learning (CNN)	Crack detection	95.1% (recall)
[335]	2019	Fei et al.	CNN (VGG-16)	Crack detection	85.9% (mIoU)
[339]	2020	Li et al.	VGG + Inception	Crack detection	95.8% (accuracy)
[113]	2020	Liu et al.	YOLOv3 + U-Net (ResNet-32)	Crack detection	95.75% (F1 score)
[355]	2020	Ibragimov et al.	Faster R-CNN	Crack detection	78.88% (AP)
[342]	2020	Zhang et al.	ALPCNet (Mask R-CNN and AFFM)	Crack detection	93.53% (F1 score)
[332]	2020	Ren et al.	DCNN	Crack detection	99.12% (accuracy)
[356]	2020	Huyan et al.	U-Net	Crack detection	99.01% (accuracy)
[341]	2020	Yamane and Chun	Mask R-CNN	Crack detection	99.15% (accuracy)
[338]	2020	Li and Zhao	CedNet (DenseNet-121)	Crack detection	98.9% (accuracy)
[343]	2020	Choi and Cha	SDDNet	Crack detection	88.0% (mIoU) 84.6% (mIoU)
[357]	2020	Feng et al.	SegNet	Crack detection	66.76% (IoU)
[358]	2020	Dong et al.	U-Net-ResNet with PAM	Crack detection	96.3% (accuracy)
[77]	2021	Nyugen et al.	CNN	Detection of road defects	> 91% (F1 score)
[359]	2021	Zhou et al.	Canny algorithm, decision tree heuristic	Crack detection	88% (Accuracy)
[112]	2022	Kortmann et al.	YOLOv4-Tiny, YOLOv4-CSP for road damage detection; VAE for severity classification	Road damage detection	42.1% (mAP for Tiny) 51% (mAP for CSP) for road damage detection, 80% for severity classification
[360]	2022	Sun et al.	DMA-Net (enhanced DeepLabv3+ model)	Crack detection and segmentation	Crack500 Dataset: 69.5% (precision), 80.0% (recall), 74.4% (F1 score) - DeepCrack Dataset: 86.9% (precision), 87.1% (recall), 87% (F1 score)

**Table 11:** Autonomous Vehicle/Robot Applications Using CV Methods.

Ref.	Year	Authors	Method	Application	Performance
[367]	2003	Na and Oh	MLP, modified potential field (MPF) method	Safe and stable navigation to a specific destination in any environment; object recognition	N/A
[79]	2016	Bojarski et al.	End-to-end learning with CNN	Determining the appropriate steering angle to ensure the vehicle can stay in its lane	98% (autonomy)
[363]	2017	Kim and Park	Sequential end-to-end transfer learning	Predicting left and right ego-lanes	>80%
[82]	2017	Chen and Huang	End-to-end learning with CNN	Determining the appropriate steering angle to ensure the vehicle can stay in its lane	N/A
[368]	2017	Ozcelik et al.	RGB→HSV conversion, SVM	Detection and recognition of traffic lights	95% (accuracy in urban areas), 88% (accuracy in traffic areas)
[93]	2017	Kim and Canny	CNN, LSTM	Interpretable learning for driverless cars by visualizing causal attention; steering angle estimation	MAE btw. 1.18–4.15
[364]	2018	Maqueda et al.	ResNet18, ResNet50	Vehicle steering angle estimation	RMSE: 4.10^0^ EVA (explained variance): 0.826 for Events input
[81]	2019	Nose et al.	End-to-end learning with CNN	Determining the appropriate steering angle to ensure the vehicle can stay in its lane	Loss: ~0.3
[86]	2019	Chen et al.	Brain-inspired cognitive model with attention (CMA), CNN, RNN, attention mechanism, LSTM	(1) Determination of free space and boundaries for existing and adjacent lanes (2) Estimating distances to obstacles and vehicle behavior (3) Learning the driving behavior and decision-making process of the human driver	Precision: 98.16%, Recall: 97.51%, F1: 97.82% in urban traffic (free space detection perf.) Precision: 99.9% in highway traffic (lane boundary detection perf.)
[13]	2019	Vishal et al.	YOLO, SVM	Traffic light recognition	94% (F1 score)
[369]	2021	Khan et al.	Pre-trained MobileNetV2	Pedestrian traffic light classification	94.92% (accuracy)
[370]	2021	Fang and Cai	ResNet18 + YOLOv3, PID algorithm	Obstacle detection and target tracking	94.12% (accuracy)
[115]	2021	Benamer et al.	DL and CV	Obstacle detection; traffic sign recognition; lane-keeping and proper decision-making	N/A
[80]	2022	Farkh et al.	CNN	Estimating the appropriate steering angle to ensure the vehicle can stay in its lane	N/A
[118]	2022	Wang et al.	FPT (fusion of a transformer and a CNN)	Detection of driver distraction	99.91% (accuracy for State Farm driver- detection dataset)
[371]	2022	Wang et al.	Improved YOLOv4	Detecting and recognizing traffic lights	Detection: 97.58% (AUC for LISA dataset), 95.85% (AUC for LaRa dataset) - Recognition: 82.15% (mAP for LISA dataset) 79.97% (mAP for LaRa dataset)
[154]	2022	Marques et al.	YOLOv3 and YOLOv3_tiny	Real-time traffic sign/traffic light detection and recognition	Competition YOLOv3: 99.08% (mAP) - YOLOv3_tiny: 98.47% (mAP) - Public Road YOLOv3: 98.914% (mAP) - YOLOv3_tiny: 95.584% (mAP)
[372]	2022	Gao et al.	Channel attention and multidimensional regression loss (CAMRL)	3D object (vehicle, pedestrian, cyclist) recognition	AP_3D|R40_ E (easy), M (moderate), H (hard) Vehicle: E: 17.12, M: 11.58, H: 9.03 - Pedestrian: E: 6.04, M: 3.85, H: 3.12 - Cyclist: E: 1.82, M: 1.15, H: 1.01
[373]	2022	Cervera-Uribe and Méndez-Monroy	U19-Net	Obstacle (vehicle and pedestrian) detection	87.08% (accuracy for vehicle), 78.18% (accuracy for pedestrian)
[374]	2022	Song et al.	Real-time obstacle detection via simultaneous refinement (RODSNet)	Real-time obstacle detection	IoU 97.9% (road), 73.8% (sidewalk), 91.9% (building), 71.7% (traffic light), 78.2% (traffic sign), 79.8% (pedestrian), 94.1% (car), 84.3% (bus) 74.1% (mIoU)

**Table 12:** Other ITS Applications Using CV Methods.

Ref.	Year	Authors	Method	Application	Performance
[376]	2015	Makantasis et al.	CNN, MLP	Fully automatic tunnel inspection; detection of concrete defects in tunnels	88.6% (accuracy)
[386]	2016	Ardestani et al.	S-T map generation, noise removal, Canny edge filtering (CEF), moving-window horizontal-line detection (MWHLD)	Detection of red-light signal time from low-resolution CCTV cameras	96.83% and 100% (detection rates for starting and ending times respectively)
[377]	2017	Ramos et al.	CNN	Detection of minor road hazards	82.8% (detection rate)
[378]	2017	Sun et al.	DxNAT, CNN	Predicting non-recurring traffic jams; identifying non-recurring traffic anomalies caused by specific events	98.73% (accuracy)
[78]	2017	Chen et al.	Cascaded CNN	Defect inspection of catenary support devices	89.2% (mAP)
[375]	2018	Xue and Li	FCN	Automatic intelligent classification and detection of tunnel lining defects; tunnel inspection	95.84% (accuracy)
[387]	2018	Zaatouri and Ezzedine	YOLOv3, transfer learning	Optimization of signal phases with real-time traffic light control algorithm based on traffic flow	N/A
[388]	2019	Qi et al.	SSD	Automatic traffic volume analysis at road junctions	81% (detection: mAP@10 FPS)
[383]	2020	Garg	Haar Cascade classifiers	Drowsiness and fatigue detection	100% (accuracy)
[389]	2020	Wyk et al.	CNN + KF	Anomaly detection in autonomous and connected vehicles	99.7% (accuracy)
[379]	2021	Acharya et al.	Deep CNN + SVM	Parking occupancy detection	99.7%, 96.7% (accuracy)
[380]	2021	Pan et al.	ResNet50	Real-time winter road surface condition monitoring; snow and ice detection using traffic cameras	95.18% (accuracy)
[24]	2021	Hurtado-Gómez et al.	YOLOv3, reinforcement learning	Traffic signal control system (vehicle counting, queue detection, traffic signal time recommendation)	92.67% (avg. recall), 100% (avg. precision)
[390]	2021	Shepelev et al.	YOLOv3	Estimation of traffic flow parameters based on tracking of speed values	N/A
[391]	2021	Umair et al.	Deep simple online and realtime tracking (Deep SORT), YOLOv4	Vehicle counting; vehicle queue length estimation	82.60% (accuracy for vehicle counting), 92.67% (accuracy for queue length estimation)
[38]	2022	Ghahremannezhad et al.	YOLOv4, KF, Hungarian algorithm, trajectory conflict analysis	Real-time accident detection using traffic cameras	93.1% (accuracy)
[392]	2022	Gao et al.	COCO-pretrained Mask R-CNN (for curb lane occupancy detection), COCO-pretrained YOLOv3 (for illegal parking detection)	Data collection and analytical approach for curb lane monitoring and illegal parking impact assessment	86–96% (detection rates for parking and bus lane occupancy), 79–86% (precision/recall values for illegal parking events
[384]	2022	Guerrieri and Parla	YOLOv3	Detecting pedestrians, vehicles, and cyclists along a tram route	96–100% (detection rate)
[393]	2022	Ahmed et al.	Multi-CNN deep model (MTCNN) + Ensemble deep learning (two InceptionV3 modules)	Automatic drowsiness detection	97.1% (accuracy)
[164]	2022	Jiao and Wang	YOLOv5, KF	Vehicle detection and tracking from traffic videos; determination of traffic flows turning in different directions; estimation of vehicle speed and location	RMSE: 10 (KF), 40 (IoU-based algorithm)
[394]	2022	Rahman et al.	HOG + Linear SVM face detector, CNN	Drowsy driving and face mask detection	97.44% (accuracy in fatigue detection), 97.90% (accuracy in face mask identification)

**Table 13:** Contributions of CV Studies.

Economic	Environmental	Social
Saving fuel and time with effective traffic management Preventing daily losses in parking systems Ensuring energy savings with efficient transportation services Reducing the number of accidents (and accordingly reducing road maintenance costs and healthcare expenditures) Contributing to employment with the creation of new domains of business	Reducing harmful gas emissions and negative environmental effects through effective traffic management Reducing air pollution and noise pollution	Increasing safety in traffic Recovering the time spent in traffic by reducing travel times Improving human quality of life by reducing the number of transportation-related accidents and traffic jams Achieving a better travel experience with autonomous and connected vehicle technologies Increasing the mobility of individuals with reduced mobility Providing safer public transport services Improving security services

## Data Availability

Not applicable.
